# Monocyte biology conserved across species: Functional insights from cattle

**DOI:** 10.3389/fimmu.2022.889175

**Published:** 2022-07-29

**Authors:** Stephanie C. Talker, G. Tuba Barut, Heidi E.L. Lischer, Reto Rufener, Lilly von Münchow, Rémy Bruggmann, Artur Summerfield

**Affiliations:** ^1^ Institute of Virology and Immunology, Bern, Switzerland; ^2^ Department of Infectious Diseases and Pathobiology, Vetsuisse Faculty, University of Bern, Bern, Switzerland; ^3^ Interfaculty Bioinformatics Unit and Swiss Institute of Bioinformatics, University of Bern, Bern, Switzerland; ^4^ Institute of Parasitology, Vetsuisse Faculty, University of Bern, Bern, Switzerland; ^5^ Bucher Biotec AG, Basel, Switzerland

**Keywords:** bovine monocyte subsets, classical monocytes, intermediate monocytes, nonclassical monocytes, transcriptome, single-cell RNA sequencing (scRNA-seq), immunometabolism, cattle

## Abstract

Similar to human monocytes, bovine monocytes can be split into CD14^high^CD16^-^ classical, CD14^high^CD16^high^ intermediate and CD14^-/dim^CD16^high^ nonclassical monocytes (cM, intM, and ncM, respectively). Here, we present an in-depth analysis of their steady-state bulk- and single-cell transcriptomes, highlighting both pronounced functional specializations and transcriptomic relatedness. Bulk gene transcription indicates pro-inflammatory and antibacterial roles of cM, while ncM and intM appear to be specialized in regulatory/anti-inflammatory functions and tissue repair, as well as antiviral responses and T-cell immunomodulation. Notably, intM stood out by high expression of several genes associated with antigen presentation. Anti-inflammatory and antiviral functions of ncM are further supported by dominant oxidative phosphorylation and selective strong responses to TLR7/8 ligands, respectively. Moreover, single-cell RNA-seq revealed previously unappreciated heterogeneity within cM and proposes intM as a transient differentiation intermediate between cM and ncM.

## Introduction

With their high functional plasticity (1, 2), monocytes are a central component of the mononuclear phagocyte system (MPS). Although their delineation from *bona fide* macrophages and *bona fide* dendritic cells has proved challenging, especially in tissues, monocytes and monocyte-derived cells are now fully appreciated as a separate lineage (2–4). In blood of humans and cattle, monocytes can be subdivided into at least two different subsets based on the expression of CD14 and CD16 (5–7): classical monocytes (cM; CD14^high^CD16^-^) and nonclassical monocytes (ncM; CD14^-/dim^CD16^high^). In mice, analogous subsets can be defined by Ly6C expression (8). A third intermediate monocyte subset (intM; CD14^high^CD16^+/high^) is less well defined and has been shown to transcriptionally resemble ncM in both humans and cattle (5, 6, 9). In these two species, cM are described as the dominant subset in peripheral blood comprising about 80% of all monocytes, while ncM and intM comprise only small fractions (about 10% each) (2, 7). In mice, however, ncM (Ly6C^-^) are reported to be as frequent as cM (Ly6C^+^) in peripheral blood (8).

Classical monocytes are known for their pro-inflammatory function especially in bacterial infections (10), however the role of intM and ncM is less well described. Nonclassical monocytes are generally viewed as anti-inflammatory and vasoprotective (11), as they were found to crawl along vascular endothelium (8, 12) and sustain vascular integrity by orchestrating endothelial renewal (13). The prominent transcription of genes for endothelial adhesion in bovine ncM (7, 9) suggests a similar role in cattle. In response to TLR7 stimulation, murine ncM have been shown to recruit neutrophils to the endothelium and to clear neutrophil-induced focal necrosis (13). Also in humans, ncM were shown to be specialized in sensing nucleic acids *via* TLR7 and TLR8 (14) and are proposed to function in antiviral immunity (15). Murine ncM have furthermore been described as biased progenitors of wound healing macrophages (16).

Monocytes in general are known to be capable of antigen presentation to T cells, however whether they are as potent as dendritic cells remains controversial (1). Notably, TLR7 stimulation, but not TLR3 or TLR4 stimulation, has been shown to promote cross-presenting abilities in murine Ly6C^+^ cM (17). Bovine monocytes, particularly ncM, were reported to induce allogeneic T-cell responses *in vitro* (18).

We have previously reported pronounced transcriptomic differences between bovine cM and ncM, as determined by bulk RNA-seq and principal component analysis (9). The present study explores these transcriptomic differences in greater detail: we performed in-depth gene-by-gene analysis of bulk- and single-cell transcriptomes, as well as analyses of TLR responsiveness and metabolic activity. Taken together, our data indicate subset-specific functions in acute inflammation, antibacterial and antiviral responses, as well as in T-cell modulation, resolution of inflammation, and tissue repair. Furthermore, the unsupervised clustering of our single-cell RNA-seq data confirms the CD14/CD16-based subset definition, but also supports continuous differentiation of bovine monocyte subsets – yet another feature presumably shared with their human counterparts (19).

## Materials and methods

### Isolation of bovine PBMC

Blood of female cattle (*Bos taurus*; various breeds; aged 6.5 months to 9 years) was collected at the Clinic for Ruminants (Vetsuisse Faculty, University of Bern, Switzerland) or at the animal facility of the Institute of Virology and Immunology (Mittelhäusern, Switzerland) by puncturing the jugular vein, using citrate-based Alsever’s solution (1.55 mM of C_6_H_12_O_6_, 408 mM of Na_3_C_6_H_5_O_7_·2H_2_O, 1.078 mM of NaCl, and 43 mM of C_6_H_8_O_7_, pH 6.2) as an anticoagulant. Blood sampling was performed in compliance with the Swiss animal protection law and approved by the cantonal veterinary authority (license numbers BE102/15, BE104/17, and BE131/17).

For peripheral blood mononuclear cell (PBMC) isolation, blood was centrifuged at 1000 x *g* for 20 min (20°C), the buffy coat was collected, diluted with PBS to a ratio of 1 to 1 (room temperature), and layered onto lymphocyte separation medium (1.077 g/mL; GE Healthcare). After centrifugation (800 x *g*, 25 min, 20°C), PBMC were collected and washed twice (400 x *g*, 8 min, 4°C) with cold PBS containing 1 mM UltraPure™ EDTA (ThermoFisher). In order to remove platelets, a final washing step was performed at 250 x *g* (8 min, 4°C).

### Phenotyping of monocyte subsets by flow cytometry

Phenotyping of bovine monocyte subsets was performed with freshly isolated PBMC in 96-well. U-bottom microtiter plates (1 x 10^7^ cells per sample). Antibodies used for the two-step five-color stainings are shown in **Table 1**. Incubations were performed for 20 min at 4°C. Washing steps between incubations (400 x *g*, 4 min, 4°C) were done with Cell Wash (BD Biosciences). Prior to staining, PBMC were incubated with bovine IgG in order to block Fc receptors (50 µg/mL; Bethyl laboratories). For detection of dead cells, Live/Dead Near-IR stain (ThermoFisher) was included in the last incubation step. Compensation was calculated by FACSDiva software using single-stained samples. For each marker to be examined on monocyte subsets, a fluorescence-minus-one (FMO) control was included. Samples were acquired with a FACSCanto II flow cytometer (BD Biosciences) equipped with three lasers (405, 488, and 633 nm). At least 5 x 10^5^ cells were recorded in the “large-cell” gate.

**Table 1:** Antibodies used for flow-cytometric phenotyping of bovine monocyte subsets.

Panel 1	Antigen	Clone/Source of mAb	Detection/Source
Core	CD14	CAM66A/Kingfisher	Anti-IgM-PE/Southern Biotech
	CD16	KD1/Bio Rad	Anti-IgG2a-AF647/Molecular Probes
	CD172a	CC149/Bio Rad	Anti-IgG2b-AF488/Molecular Probes
Phenotypic marker (Pm)	CD5	AFRCIAH-CC29/in house	Anti-IgG1- BV421/BioLegend
	CD8α	CACT80C/Kingfisher	
	CD11a	HUH73A/Kingfisher	
	CD40	IL-A156/Kingfisher	
	CD43	CO.44B8/Bio-Rad	
	CD45	1.11.32/Bio-Rad	
	CD62L	Du-1-29/in house	
	CD80	IL-A159/Kingfisher	
	CD86	IL-A190A/Kingfisher	
	CD163	LND68A/Kingfisher	
	CD205	IL-A114/Bio Rad	
	MHC-II	VPM54/in house	
**Panel 2**	**Antigen**	**Clone/Source of mAb**	**Detection/Source**
Core	CD14	CAM36A/Kingfisher	Anti-IgG1-BV421/BioLegend
	CD16	KD1/Bio Rad	Anti-IgG2a-AF647/Molecular Probes
	CD172a	CC149/Bio Rad	Anti-IgG2b-AF488/Molecular Probes
Pm	CD11c	BAQ153A/Kingfisher	Anti-IgM-PE/Southern Biotech
**Panel 3**	**Antigen**	**Clone/Source of mAb**	**Detection/Source**
Core	CD14	CAM66A/Kingfisher	Anti-IgM-PE/Southern Biotech
	CD16	KD1/Bio Rad	Anti-IgG2a-AF647/Molecular Probes
	CD172a	DH59B/Kingfisher	Anti-IgG1-BV421/BioLegend
Pm	CD11b	MM10A/Kingfisher	Anti-IgG2b-AF488/Molecular Probes

### Fluorescence-activated cell sorting and bulk RNA sequencing

Bulk RNA sequencing data of sorted monocyte subsets are derived from previous experiments, described in Talker et al. (9). Experimental procedures are therefore described only briefly and the reader is referred to our previous publication for more details. In order to sort bovine monocyte subsets for Illumina sequencing, a two-step staining was performed with 3 x 10^8^ freshly isolated PBMC. Classical monocytes (cM) were sorted as CD172a^high^CD14^high^CD16^-^, intM as CD172a^high^CD14^high^CD16^high^ and ncM as CD172a^high^CD14^-/dim^CD16^high^ using a FACS Aria (BD Biosciences). All sorted subsets had a purity of at least 97%. Per subset, at least 1 x 10^5^ sorted cells were frozen in TRIzol (ThermoFisher) for later RNA extraction (Nucleospin RNA kit, Macherey Nagel). High-quality RNA (approximately 500 ng; RIN> 8) was used for nondirectional paired-end mRNA library preparation (TruSeq Sample Preparation Kit; Illumina).

Sequencing was performed on the Illumina HiSeq3000 platform using 100 bp single-end sequencing, yielding between 25.2 and 41.1 million read pairs per sample. Reads were mapped to the *Bos taurus* reference genome (UMD3.1) with Hisat2 v. 2.1.0, and FeatureCounts from Subread v. 1.5.3 was used to count the number of reads overlapping with each gene, as specified in the Ensembl annotation (release 91). Raw counts of the sequencing data previously published in Talker et al., 2018 (9) were re-analyzed with the Bioconductor package DESeq2 (20), including only data for the three monocyte subsets and considering the factor “animal” in the design formula. Raw counts were normalized to account for differences in sequencing depth between samples. Gene length was not considered. Lists of differentially expressed genes were obtained by performing pairwise comparisons with DESeq2 (adjusted p-value < 0.05). Gene lists were manually screened for genes of interest using the human gene database GeneCards^®^ and literature research *via* PubMed^®^. Principal component analysis was performed with normalized and rlog-transformed counts of the 1000 most variable genes across monocyte samples. Heatmaps were prepared following log2 transformation of normalized counts. Prior to log2 transformation, a pseudocount of 1 was added to the values to avoid zeros. All analyses were performed using R version 3.6.1.

For gene set enrichment analysis (GSEA) we used genes ranked based on the DESeq2 output (cM vs. ncM, filtered for p_adj < 0.05) according to the “stat” value representing the Wald statistics. We employed the GSEA software from UC San Diego and Broad Institute (21, 22) with the C5:GO:BP gene sets (Gene Ontology, biological process, file c5.go.bp.v7.5.1.symbols.gmt) that are integrated into the software (MSigDB v7.4) (22, 23). The following parameters were used: gene set size “15-500”; scoring scheme “weighted”, normalization “meandiv”, mode “mean of probes”, number of permutations “1000”.

The bulk-RNA-seq datasets are available in the European Nucleotide Archive (http://www.ebi.ac.uk/ena) under the accession number PRJEB28324.

### Phosphoflow cytometry

The following TLR ligands were used at a final concentration of 10 μg/mL to assess TLR responsiveness of bovine monocyte subsets: Pam2CSK4 [C_65_H_126_N_10_O_12_S • 3TFA] (InvivoGen), high-molecular-weight (HMW) polyinosinic-polycytidylic acid [Poly(I:C)] (InvivoGen), LPS from *E. coli* strain K12 [LPS-EK] (InvivoGen), Gardiquimod (Sigma-Aldrich), Resiquimod (Sigma-Aldrich).

Stimulation with TLR ligands and staining for phosphorylated p38 MAPK was performed as reported previously (24). Prior to stimulation, cell surface staining was performed. Following Fc-receptor blocking with purified bovine IgG (50 µg/mL; Bethyl laboratories), defrosted and CD3-depleted bovine PBMC were stained with anti-CD172a (CC149, IgG2b), anti-CD14 (CAM36A, IgG1), and anti-CD16 (KD1, IgG2a), followed by incubation with anti-IgG2b-AF488 (Molecular Probes), anti-IgG1-biotin (Southern Biotech) and anti-IgG2a-PE (Southern Biotech). In a fourth step, ChromPure mouse IgG (Jackson ImmunoResearch) was added together with Streptavidin-BV421 (BD Biosciences) and Live/Dead™ Fixable Near-IR stain (Thermo Fisher Scientific). Stained cells were incubated for 15 minutes with respective TLR ligands in PBS or with PBS alone (waterbath at 37°C). Immediately after this incubation period, cells were fixed with BD Cytofix/Cytoperm™ fixation buffer (BD Biosciences; 12 min at 37°C) and thereafter stained with anti-p38 MAPK-AF647 (BD Phosflow; 30 min at 37°C). Samples were acquired with a FACSCanto II flow cytometer (BD Biosciences) equipped with three lasers (405, 488, and 633 nm). At least 1.5 × 10^6^ cells were recorded in the “large-cell” gate. Compensation was calculated by FACSDiva software using single-stained samples.

### Extracellular flux analysis

For metabolic assays using a Seahorse Extracellular Flux Analyzer (denominated “Seahorse assays”; Agilent Technologies Inc.), bovine monocyte subsets were FACS-sorted from magnetically enriched CD172a^+^ PBMC. Combined staining for magnetic selection and FACS was performed in 50 mL centrifugation tubes and included five incubation steps, each carried out at 4°C for 20 min. Staining, and washing (400 x g, 8 min, 4°C) in-between each incubation step, was performed in PBS containing 1 mM EDTA and 5% (v/v) heat-inactivated FBS (GIBCO, Life Technologies). In a first step, freshly isolated PBMC (4 x 10^8^) were incubated with bovine IgG (50 µg/mL; Bethyl laboratories) to block Fc receptors. This was followed by incubation with the primary antibodies anti-CD172a (CC149, IgG2b), anti-CD14 (CAM36A, IgG1), and anti-CD16 (KD1, IgG2a) and the secondary antibodies anti-IgG1-AF647 (Molecular Probes) and anti-IgG2a-PE (Southern Biotech). In a fourth step, anti-mouse IgG magnetic beads (Miltenyi Biotec) were added, and cells were loaded onto two LS columns (Miltenyi Biotec) for magnetic enrichment of CD172a expressing cells. In a fifth step, anti-IgG2b-AF488 (Molecular Probes) was added, resulting in a dim staining of CD172a for FACS. Enriched monocytes were sorted on a MoFlo Astrios EQ cell sorter (Beckman Coulter) equipped with five lasers at the Flow Cytometry and Cell Sorting Facility (FCCS) of the University of Bern. Purity of subsets was confirmed by re-analysis of samples and was shown to be at least 98%. Following sorting, cells were resuspended in pH-optimized Seahorse assay medium (Agilent Seahorse XF DMEM Medium supplemented with 10 mM Agilent Seahorse XF Glucose, 1mM Agilent Seahorse XF Pyruvate, and 2mM Agilent Seahorse XF Glutamine) and 5 x 10^5^ cells (*ex vivo*) or 1 x 10^5^ cells (after 6-day culture) were seeded in duplicates or triplicates into an 8-well Seahorse plate (Agilent Seahorse XFp FluxPak). Two wells served as background controls. During manual cell counting, viability of sorted subsets was confirmed to be above 95% by trypan blue staining. Cells not immediately used for Seahorse assays, were incubated for six days at 37°C (5% CO_2_) to allow for differentiation into monocyte-derived macrophages. Specifically, remaining cells were seeded into a 12-well plate with 1 x 10^6^ cells per well in 2 mL culture medium consisting of DMEM-GlutaMAX™ (Gibco, ref.no: 31966-021), supplemented with penicillin (50 I.U./mL), streptomycin (50 µg/mL), 10% heat-inactivated FBS, and 1% M-CSF (produced in house; titrated to optimize viability of monocytes in culture). After six days of incubation, cells were harvested, resuspended in Seahorse assay medium, counted, and processed as described above. Viability of harvested cells was above 90% as assessed microscopically by the trypan blue exclusion test.

Seahorse preparations and measurements were performed according to the manufacturer’s instructions. For measurements with the Agilent Seahorse XFp Analyzer, the standard settings of the XFp Real-Time ATP Rate Assay were used. Following basal measurements, oligomycin (final concentration 9 µM), an inhibitor of ATP synthase (complex V of the electron transport chain), was added. After another measurement phase, a mastermix of rotenone (final concentration 8 µM), an inhibitor of complex I, and antimycin A (final concentration 8 µM), an inhibitor of complex III, was added. Inhibitors of the electron transport chain were purchased from Sigma-Aldrich (cat. no. O4876, R8875, A8674) and titrated on bovine cM to determine optimal concentrations. For establishment of the procedure, Agilent Seahorse XFp Real-Time ATP Rate Assay Kit (cat. no. 103591-100) was used according to manufacturer’s instructions, but with an increased final concentration (6 µM) of the provided oligomycin, according to prior titration on bovine cM. Data were analyzed using the Seahorse Wave Desktop Software version 2.6.1 (Agilent).

### Single-cell RNA sequencing (10x Genomics)

For scRNA-seq, PBMC from two cows (6.5 months old; #CH4431 and #CH4432) were isolated as described above. Cell counting and viability assessments were carried out microscopically using the trypan blue exclusion test. Thereafter, cells were delivered to the Next Generation Sequencing Platform at the University of Bern and processed as follows: GEM generation & barcoding, reverse transcription, cDNA amplification and 3’ gene expression library generation steps were all performed according to the Chromium Single Cell 3’ Reagent Kits v3 User Guide (10x Genomics CG000183 Rev B) with all stipulated 10x Genomics reagents. Generally, 16.0 µL of each cell suspension (600-800 cells/µL) and 30.6 µL of nuclease-free water were used for a targeted cell recovery of 10’000 cells. GEM generation was followed by a GEM-reverse transcription incubation, a clean-up step and 11 cycles of cDNA amplification. The resulting cDNA was evaluated for quantity and quality using a Thermo Fisher Scientific Qubit 3.0 fluorometer with the Qubit dsDNA HS Assay Kit (Thermo Fisher Scientific, Q32851) and an Advanced Analytical Fragment Analyzer System using a Fragment Analyzer NGS Fragment Kit (Agilent, DNF-473), respectively. Thereafter, 3′ gene expression libraries were constructed using a sample index PCR step of 16 cycles. The generated cDNA libraries were tested for quantity and quality using fluorometry and capillary electrophoresis as described above. The cDNA libraries were pooled and sequenced with a loading concentration of 300, paired end and single indexed, on an illumina NovaSeq 6000 sequencer using a NovaSeq 6000 SP Reagent Kit v1 (100 cycles; illumina, 20027464). The read set-up was as follows: read 1: 28 cycles, i7 index: 8 cycles, i5: 0 cycles and read 2: 91 cycles. The quality of the sequencing runs was assessed using illumina Sequencing Analysis Viewer (illumina version 2.4.7) and all base call files were demultiplexed and converted into FASTQ files using illumina bcl2fastq conversion software v2.20. More than 36’000 reads/cell were generated for each sample.

Mapping and counting of UMIs was performed using Cell Ranger (version 3.0.2, 10x Genomics) with the reference genome ARS-UCD1.2 from Ensembl to build the necessary index files. Subsequent analysis was performed in R (version 4.0.2)  (25). The Scater package (version 1.16.2)  (26) was used to assess the proportion of ribosomal and mitochondrial genes as well as the number of detected genes. Cells were considered as outliers and filtered out if the value of the proportion of expressed mitochondrial genes or the number of detected genes deviated more than three median absolute deviations from the median across all cells. Additionally, all ribosomal genes were removed. After quality control, the sample from cow #CH4431 retained 6165 cells and the sample from cow #CH4432 retained 6401 cells. Normalization between samples was done with the deconvolution method of Lun et al.  (27) using the package Scran (version 1.16.0)  (28). Samples were integrated with the FindIntegrationAnchors function of the package Seurat (version 3.2.0) based on the first 30 principal components (PCs)  (29). Graph-based clustering was done with the FindNeighbors and FindClusters functions of the Seurat package using the first 35 PCs from the dimensionality reduction step. The Clustree package (version 0.4.3)  (30) was used to determine the resolution (0.8) resulting in clustering concurring with the presumed cell types. Clusters were annotated based on marker genes that were identified with the FindAllMarkers function of Seurat. Cells from clusters identified as monocytic cells (c4, c10, c14, and c18) were extracted and re-clustered in an identical fashion as above, but with a resolution of 0.6. The scRNA-seq datasets are available in the European Nucleotide Archive (http://www.ebi.ac.uk/ena) under the accession number PRJEB50632.

The R package Monocle3 (31–33) was used to do a trajectory analysis on the clusters 0-4 of the re-clustered cells. UMAP dimensionality reduction method was used and the Louvain clustering method using 100 nearest neighbors (function cluster_cells of Monocle3). The function graph_test of Monocle3 was used to find genes that are differentially expressed across a single-cell trajectory. Default parameters were used unless stated otherwise.

### Ethics Statement

The animal experiments were performed in compliance with the Swiss animal protection law (TSchG SR 455; TSchV SR 455.1; TVV SR 455.163). The procedures were reviewed by the committee on animal experiments of the canton of Bern, Switzerland, and approved by the cantonal veterinary authority (Amt für Landwirtschaft und Natur LANAT, Veterinärdienst VeD, Bern, Switzerland) under the license numbers BE102/15, BE104/17, and BE131/17.

### Preparation of figures

Figures were prepared using FlowJo version 10 (FlowJo LLC, Ashland, OR), GraphPad Prism versions 7.0.3 and 9.3.1 for Windows (GraphPad Software, San Diego, CA), R version 3.6.1., and Inkscape (www.inkscape.org).

### Statistical analysis

Fold change in median fluorescence intensity (MFI; phosphoflow cytometry) was tested for statistical significance using multiple paired t-tests on log-transformed MFI values of PBS-incubated versus stimulated samples. Obtained p-values are shown alongside q values (two-stage step-up, Benjamini, Krieger, and Yekutieli; desired FDR 5%) and adjusted p-values (Holm-Šídák method).

Differences in glycolytic and mitochondrial ATP production rates (Seahorse assays) were tested for statistical significance using multiple paired t-tests on means of duplicates/triplicates across four assays. Adjusted p-values were determined by the Holm-Šídák method. Calculations were done using GraphPad Prism version 9.3.1 for Windows.

## Results

### Phenotype and transcriptome of nonclassical and intermediate monocytes differ markedly from classical monocytes

**Figure 1:**
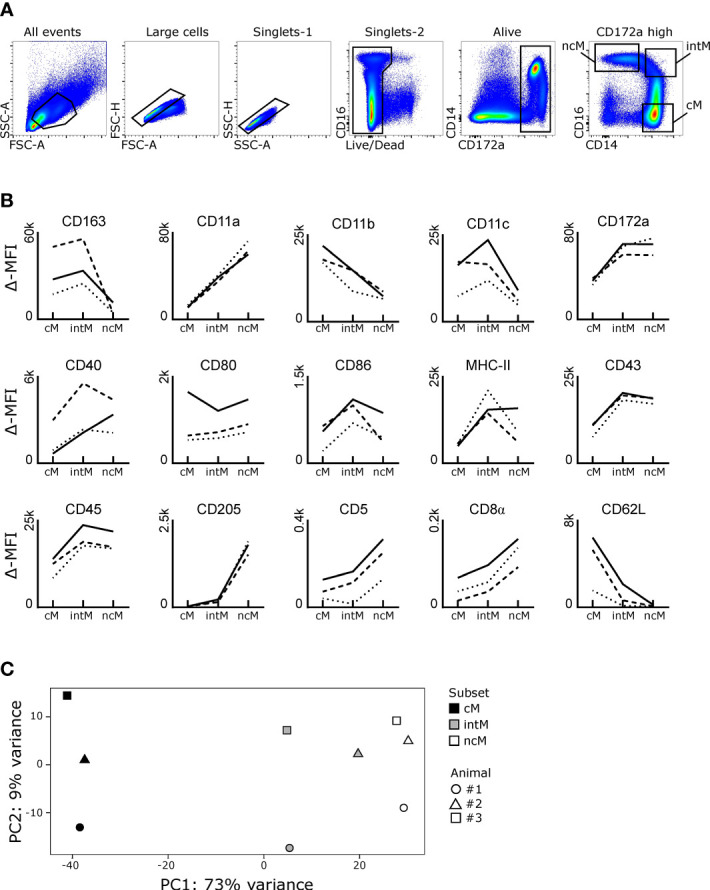
Phenotype and transcriptional clustering of bovine monocyte subsets. **(A, B)** Flow cytometric analysis. Freshly isolated PBMC were stained for flow cytometry. Monocyte subsets were gated based on expression of CD14 and CD16 within CD172a^high^ cells after gating on large cells (FSC^high^), single cells (within diagonal in FSC-A vs. FSC-H and SSC-A vs. SSC-H), and living cells (Near-IR^low^). Classical monocytes (cM) were gated as CD14^high^CD16^-^, intermediate monocytes (intM) as CD14^high^CD16^high^, and nonclassical monocytes (ncM) as CD14^-/dim^CD16^high^. **(B)** Graphs show the delta median fluorescence intensity (MFI) of surface expression for selected molecules. Delta MFI was calculated as the difference in MFI between stained samples and FMO controls. Lines illustrate the delta MFI across monocyte subsets. Stainings were performed on seven different animals, resulting in 3 animals analyzed per marker. Within single graphs, data of three different animals is illustrated by solid, dashed, and dotted lines. **(C)** First two axes of a principal component analysis (PCA) including the 1000 most variable genes. Illumina sequencing was performed on RNA isolated from sorted monocyte subsets of three animals. Each dot represents one sample, with the color coding for different cell subsets and the shape coding for the three different animals.

Bovine monocyte subsets were analyzed by flow cytometry for expression of various surface molecules (**Figures 1A, B**). While some molecules showed clear subset-dependent expression patterns (e.g. CD163, CD11a, CD11b, CD205), other molecules varied strongly with the animals analyzed (e.g. CD40, CD80, MHC-II). A gradual increase from cM over intM to ncM could be observed for expression of CD11a, whereas a gradual decrease was observed for CD11b. In addition to CD11a, ncM expressed the highest levels of CD5, CD8α, and CD205. Intermediate monocytes expressed the highest levels of CD86 and, for 2 out of 3 animals, also expressed the highest levels of MHC-II. Highest expression on intM combined with lowest expression on ncM was seen for CD163 and CD11c. Expression of CD172a, CD45 and CD43 was higher on ncM and intM compared to cM. Furthermore, cM expressed the highest levels of CD62L, being almost absent from ncM. **Supplementary File 1** illustrates exemplary flow cytometry data as well as marker expression on CD14^high^CD16^dim^ monocytes, which were not included in any sorting gates for bulk RNA-seq in the present study. These cells showed expression levels in-between CD14^high^CD16^-^ (cM) and CD14^high^CD16^high^ (intM) monocytes for most markers analyzed, except for CD163, CD11b and CD11c, which were expressed on CD14^high^CD16^dim^ cells at levels at least as high or even higher than on cM of the same animal.

Taken together, our phenotypic analyses have confirmed previously described expression patterns of MHC-II, CD172a, CD62L, CD11a and CD11b on bovine monocyte subsets (7) and provide new information on the expression of CD45, CD43, CD5, CD40, CD80, CD86, CD11c, CD163, and CD205. Moreover, in contrast to a previous study, where CD8α expression was concluded to be absent from all monocytes (34), we found indications of weak CD8α expression on ncM and intM. A recent paper, citing the pre-print version of the present manuscript (35), summarizes phenotypic characteristics of bovine monocyte subsets and gating strategies (36).

Principal component analysis of previously published bulk RNA-seq data (9) revealed that differences between monocyte subsets explained the highest proportion of total variance (73%, PC1) in the transcriptomic dataset, followed by 9% of variance (PC2) explained by differences between animals (**Figure 1C**). As expected from previous analyses (9), ncM and intM clearly clustered apart from cM, with intM clustering much closer to ncM than to cM. Notably, when looking at PC1, intM of animal #2 clustered closer to ncM samples than to intM samples of the other two animals.

Identity of monocyte subsets was confirmed by subset-specific transcription of the key genes *NR4A1* (ncM, intM), *CX3CR1* (ncM, intM) and *CCR2* (cM) (**Supplementary File 2A**). Moreover, the transcription of surface molecules previously analyzed by flow cytometry in different animals followed the same patterns. Except for CD163, for which mRNA content in intM was lower than expected, and CD11b *(ITGAM*) and CD43, for which mRNA content in ncM was higher than in intM (**Supplementary File 2B**). Diverging patterns were also observed for CD80, with considerable individual variation both on mRNA and protein level.

Pairwise comparisons of monocyte subsets revealed a variety of differentially expressed genes, involved in various immune functions. The genes addressed in the following chapters have been selected based on pairwise comparisons (DESeq2; adjusted p-value < 0.05) and literature research, and raise no claim for completeness. The output from pairwise comparisons, including normalized counts for all genes and subsets, is provided in **Supplementary File 3**.

### Pro-inflammatory gene expression prevails in classical monocytes

**Figure 2:**
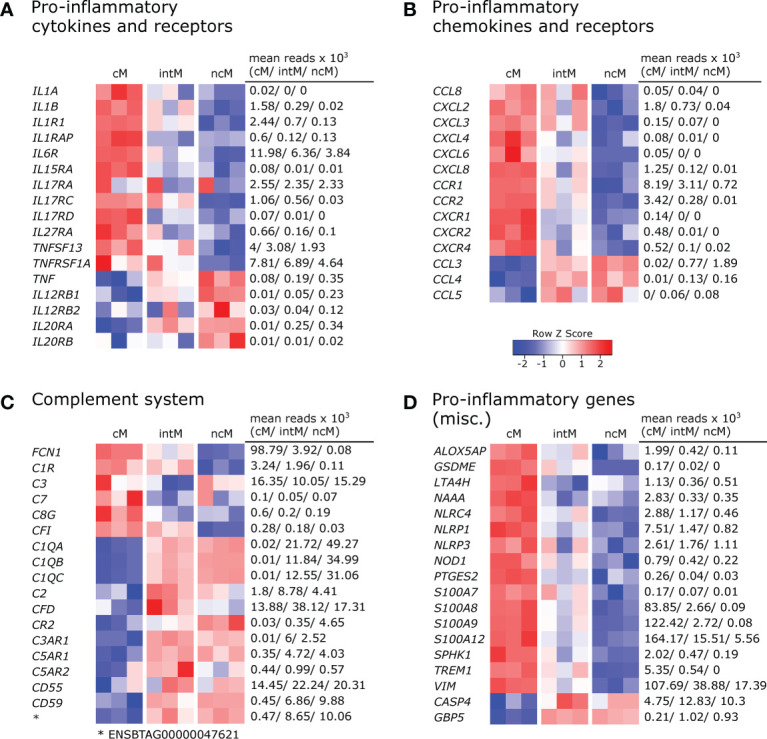
Pro-inflammatory gene expression. Illumina sequencing was performed on RNA isolated from sorted monocyte subsets (cM, intM, ncM) of three animals. Heatmaps show row z-scores calculated from log2-transformed normalized counts of selected genes coding for pro-inflammatory cytokines and receptors **(A)**, pro-inflammatory chemokines and receptors **(B)**, proteins associated with the complement system **(C)**, and other pro-inflammatory mediators **(D)**. Mean kilo reads for each subset and gene are given to the right of each heatmap. Genes were selected based on pairwise comparisons with DESeq2 (adjusted p-value < 0.05) and literature research.

Monocyte subsets clearly differed in the transcription of inflammatory cytokines and cytokine receptors (**Figure 2A**). Classical monocytes were strongly enriched in transcripts for IL-1 (*IL1A*, *IL1B*) and for the IL-1 receptor (*IL1R1*, *IL1RAP*). Moreover, cM predominantly expressed *IL6R*, *IL15RA*, *IL17RA*, *IL17RC*, *IL17RD* and *IL27RA*. Trans-signaling with soluble IL-6 receptor is reported to mediate pro-inflammatory functions of IL-6 (37) and also *in vitro* stimulation with IL-27 is reported to increase inflammasome activation in monocytes (38). Furthermore, expression of *TNFSF13* (APRIL), reported to induce IL-8 production in the human macrophage-like cell line THP-1 (39) and *TNFRSF1A* (TNFR1), mediating pro-inflammatory signaling of TNF-α, was highest in cM. Notably, *TNF* was found to be primarily expressed in ncM and intM. Tumor necrosis factor alpha (*TNF*/TNF-α) is regarded as a master regulator of inflammation with various effects on different cell types. TNF-α is produced either in soluble form which signals mainly *via* TNFR1 (*TNFRSF1A*), or as transmembrane protein, being the main ligand for TNFR2 (*TNFRSF1B*) (40) and implicated in reverse signaling (41), the functional consequences of which are incompletely understood (41, 42). Moreover, receptors for IL-12 and IL-20 (*IL12RB1*, *IL12RB2* and *IL20RA*, *IL20RB*), were mainly expressed in ncM, though reads for *IL20RB* were very low (mean=20). Although little is known about IL-12-receptor- and IL-20-receptor signaling in monocytes, *in vitro* studies suggest overall pro-inflammatory effects (43, 44).

Also a number of pro-inflammatory chemokines and chemokine receptors were found to be differentially expressed among monocyte subsets (**Figure 2B**). Looking at chemokines overexpressed in cM, the most pronounced differences between cM and ncM were found in the expression of *CXCL2* and *CXCL8*. Moreover, transcripts for *CCL8*, *CXCL3*, *CXCL4* and *CXCL6* could be detected in cM and in intM of two animals, though all with low number of reads (max. 150 mean reads). Chemokine receptors associated with inflammation were all predominantly expressed by cM. Expression of *CCR1* in cM was upregulated 2-fold over intM and 11-fold over ncM. Expression of *CCR2* and *CXCR4* was clearly upregulated in cM over intM (12-fold and 5-fold, respectively), and almost absent from ncM. Exclusive expression in cM was observed for *CXCR1* and *CXCR2*. Nonclassical monocytes and intM clearly showed the highest expression of *CCL3* and were also enriched in transcripts for *CCL4* and *CCL5*, though at a lower level.

Gene expression also supports a prominent role of cM in complement-mediated inflammatory processes (**Figure 2C**). We found that transcription of *FCN1*, a recognition receptor for the lectin complement pathway, was highly increased in cM and almost absent in ncM, as was the transcription of *C1R*, a subunit of the complement C1. Notably, transcripts for complement component C3, which is central for activation of both the classical and the alternative complement pathway, were enriched in cM and ncM, and showed the lowest levels in intM, whereas complement factor I (*CFI*, C3b-Inactivator), an important negative regulator of both complement pathways, was exclusively transcribed in cM and intM. Moreover, transcription of *C7* as well as of *C8G*, both involved in formation of the membrane attack complex, was highest in cM. Nevertheless, certain important genes of the complement system were overexpressed in both ncM and intM. Among those genes were *C1QA*, *C1QB*, and *C1QC*, all of which were barely expressed in cM. The molecule C1q is one of the main sensors of PAMPs and DAMPs, but also antibody complexes, in the classical complement pathway and has been associated with tolerogenic functions (45). Notably, C1q also binds to the surface of dead cells, thereby promoting their phagocytosis. Complement factor 2 (*C2*) and complement factor D (*CFD*), involved in the classical and alternative complement pathway, respectively, showed the highest transcription in intM. The receptor for complement factor C3a (*C3AR1*) was exclusively expressed in intM and ncM, whereas transcription of the receptor for complement factor C3d (*CR2*/CD21) was markedly increased in ncM and to a lesser extent in intM, when compared to cM. Both known receptor genes for complement factor C5a, *C5AR1* (CD88) and *C5AR2,* showed increased expression in ncM and intM, with slightly higher expression in intM. While C5aR1 is regarded as a mediator of pro-inflammatory signaling, C5aR2 has recently received attention as a multifaceted modulator of C5a signaling, described to dampen inflammasome activation and to alter TLR signaling (46). Furthermore, we found a higher transcription of *CD55* in ncM/intM compared to cM, encoding a complement inhibitory protein reported to suppress T-cell responses (47). A 20-fold increased transcription in ncM as compared to cM was found for *CD59*, encoding a receptor for C8 and C9. Apart from its function as an inhibitor of the membrane attack complex, CD59 expression on antigen-presenting cells was reported to deliver suppressive signals to murine CD59-expressing CD4 T cells *via* a complement-independent ligand (48, 49). Nonclassical monocytes were also clearly enriched in transcripts for the gene ENSBTAG00000047621. A protein query revealed the highest similarity with human (53.7%) and murine (47.5%) C4BPA, a molecule implicated in the inhibition of classical complement activation that has been reported to induce an anti-inflammatory state in monocyte-derived dendritic cells (50). However, no transcripts were found for the gene annotated as bovine C4BPA.

Also transcription of other genes associated with pro-inflammatory functions was clearly dominant in cM (**Figure 2D**). This includes genes coding for sensory components of inflammasomes (*NOD1*, *NLRC4*, *NLRP1*, *NLRP3*), pyroptosis-mediating gasdermin E (*GSDME*), enzymes involved in the biosynthesis of leukotriens (*ALOX5AP*, *LTA4H*) and prostaglandins (*PTGES2*), as well as the kinase for generation of sphingosine-1-phosphate (*SPHK1*), the pro-inflammatory receptor TREM1, the pro-inflammatory amidase NAAA (51, 52), and S100 proteins promoting inflammation (*S100A7*, *S100A8*, *S100A9*, *S100A12*) (53). Furthermore, transcripts for vimentin (*VIM*), reported to be a key positive regulator of the NLRP inflammasome (54), were clearly enriched in cM. While none of the monocyte subsets contained transcripts for nitric-oxide synthases at steady state (data not shown), we could recently show that bovine cM massively increase transcription of *NOS2* upon *in vitro* stimulation with TLR ligands (24). Nonclassical monocytes expressed the highest levels of *CASP4* and *GBP5*, the latter being described as an activator of inflammasome assembly (55). Caspase 4 (*CASP4*), being part of the non-canonical inflammasome, is described to promote pro-inflammatory cytokine production, but recently has also been implicated in autophagy (56) – a process that may negatively regulate inflammasome signaling. Taken together, the differential expression of pro-inflammatory genes suggests fundamentally different functions of bovine monocyte subsets.

### Gene expression and TLR responsiveness indicate complementary functions of cM and ncM in antibacterial and antiviral immunity

**Figure 3:**
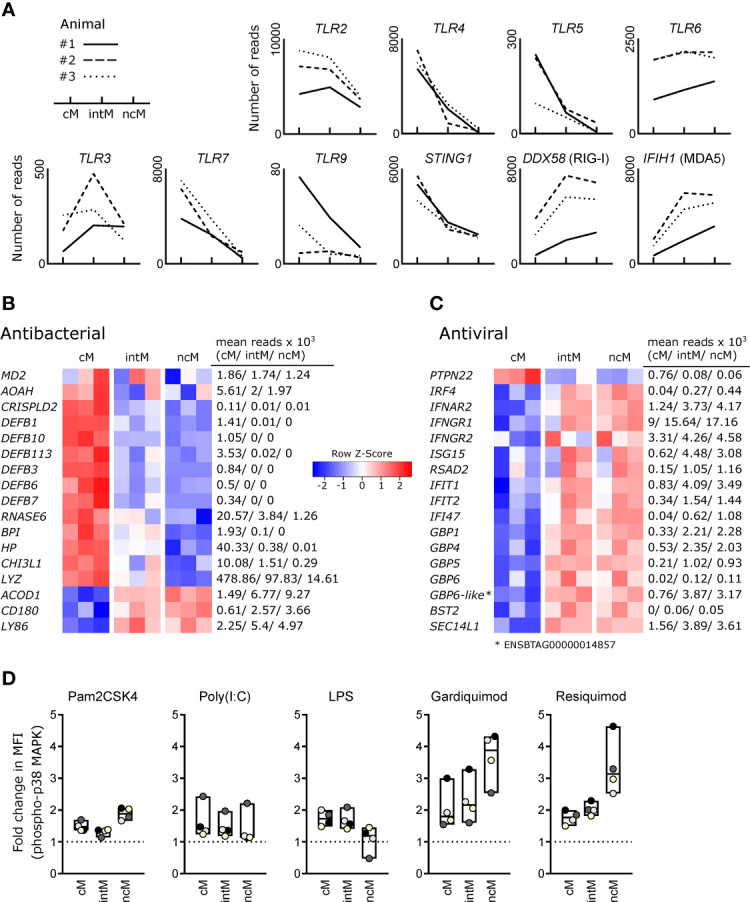
Antimicrobial gene expression and TLR responsiveness. Illumina sequencing was performed on RNA isolated from sorted monocyte subsets (cM, intM, ncM) of three animals. **(A)** Gene expression for pattern-recognition receptors. Graphs show the number of reads across monocyte subsets for selected genes with individual animals indicated by solid (#1), dashed (#2) and dotted (#3) lines. **(B, C)** Heatmaps show row z-scores calculated from log2-transformed normalized counts for genes associated with antibacterial **(B)** and antiviral **(C)** responses. Mean kilo reads for each subset and gene are given to the right of each heatmap. Genes were selected based on pairwise comparisons with DESeq2 (adjusted p-value < 0.05) and literature research. **(D)** Responsiveness of bovine monocyte subsets to TLR ligands. Defrosted bovine PBMC were depleted of CD3^+^cells, stained for CD172a, CD14 and CD16, and stimulated with Pam2CSK4, Poly(I:C), LPS, Gardiquimod, or Resiquimod for 15 min, before being fixed/permeabilized and stained with a fluorochrome-conjugated monoclonal antibody against phosphorylated p38 MAPK. Incubation with PBS served as control. Graphs show the fold change in median fluorescence intensity (MFI of stimulated sample divided by MFI of PBS control) of phospho-p38 MAPK staining for cM (CD14^high^CD16^-^), intM (CD14^high^CD16^high^) and ncM (CD14^-/dim^CD16^high^). For each stimulation, data of four different animals (color-coded dots) is shown. Boxes indicate minimum, maximum and median values. Paired t-tests were performed on log-transformed MFI values of stimulated samples vs. PBS-incubated samples. Statistical analyses and exemplary flow cytometry plots are given in **Supplementary File 4**.

Looking at the gene expression of pattern recognition receptors, we found that *TLR2* was expressed higher in cM and intM compared to ncM, and that *TLR4* and *TLR5* transcripts were clearly enriched in cM (**Figure 3A**). Toll-like receptor 6 (*TLR6*) showed a trend towards higher expression in intM and ncM. Expression of *TLR3* differed markedly between animals, but was in tendency highest in intM. Furthermore, cM expressed the highest levels of *TLR7* and *STING1*, the latter encoding a cytoplasmic receptor for DNA of both viral and bacterial origin. For two out of three animals, *TLR9* expression was also highest in cM. Transcript levels for RIG-1 (*DDX58*) and MDA-5 (*IFIH1*), however, were higher in ncM and intM.

Bovine cM were clearly enriched in transcripts involved in antibacterial responses (**Figure 3B**). These transcripts encode an accessory protein for TLR4 (*MD2*), other LPS-binding proteins [*AOAH* (57), *CRISPLD2* (58)], beta-defensins (*DEFB1*, *DEFB3*, *DEFB6*, *DEFB7*, *DEFB10*, *DEFB113*) (59), and other proteins commonly known or described to be involved in antibacterial responses (*RNASE6* (60), *BPI* (61, 62), *HP* (63), *CHI3L1* (64), *LYZ*). Notably, high levels of *MD2* were also expressed by intM of two animals. Three genes associated with antibacterial responses were found to be upregulated in ncM and intM – *ACOD1*, *CD180* and *LY86* (MD1), the latter two genes coding for LPS-binding proteins and members of the TLR family that form a complex to regulate TLR4 signaling (65). ACOD1 (IRG1) mediates the production of itaconate, which is – apart from its anti-inflammatory functions – also known for its antibacterial properties (66).

Looking at genes associated with antiviral responses, we found one gene upregulated in cM (*PTPN22*) and the vast majority of genes upregulated in intM and ncM (**Figure 3C**). PTPN22, overexpressed in cM, has been reported to potentiate TLR-induced type-I interferon production (67), and to regulate inflammasome activation (68). Both ncM and intM clearly expressed the highest levels of *IRF4* and *IFNAR2*, whereas *IFNAR*1 was expressed to similar levels in all monocyte subsets (data not shown). Also *IFNGR1* and *IFNGR2*, coding for the IFN-γ receptor, showed increased transcription in ncM and intM. Accordingly, the transcription of interferon-induced antiviral genes (*ISG15*, *RSAD2*, *IFIT1*, *IFIT2*, *IFI47*) was higher in ncM and intM, as compared to cM. Notably, the ubiquitin-like protein ISG15 exerts its antiviral function intracellularly by ISGylation of viral proteins and also extracellularly by acting in a cytokine-like manner to promote IFN-γ production of NK cells and T cells (69). Viperin (*RSAD2*), a multifunctional antiviral factor (70), has recently gained attention, as it was shown to act as a synthase for antiviral ribonucleotides (71).

Moreover, ncM and intM were enriched in transcripts for several interferon-induced guanylate-binding proteins (GBP1, GBP4, GBP5, GBP6, ENSBTAG00000014857). Alongside the GBP4 gene displayed in the heatmap (ENSBTAG00000037634), also three other genes annotated as GBP4 (ENSBTAG00000014529, ENSBTAG00000038233) or GBP4-like (ENSBTAG00000002416) were upregulated in ncM and intM (data not shown). Only recently, GBP1 has been allocated an important role in apoptosis and pyroptosis of human macrophages (72). GBP4 has been reported to negatively regulate virus-induced type I IFN responses by targeting interferon regulatory factor 7 (73). Along this line, BST2, exclusively expressed in ncM and intM, was reported to inhibit type I interferon and cytokine production in TLR7/9-stimulated pDC (74). Furthermore, SEC14L1, more than 2-fold enriched in ncM and intM, was reported to negatively regulate RIG-I-mediated signaling (75).

Responsiveness of monocyte subsets to TLR stimulation, as determined by phosphoflow cytometry for p38 MAPK (**Figure 3D**), corroborates a specialization of cM and ncM for antibacterial and antiviral responses, respectively. While the TLR4 ligand LPS induced responses primarily in cM and intM, stimulation with the TLR7/8 ligands Gardiquimod and Resiquimod induced the strongest responses in ncM. This may point towards high TLR8 expression in ncM (*TLR8* not annotated), as transcript levels for *TLR7* were relatively low in ncM. Also responses to Pam2CSK4, a synthetic diacylated lipopeptide and ligand for TLR2/6, were highest in ncM. At last, similar low-level responses to Poly(I:C) were detectable in all subsets, with one animal standing out by a considerably higher response (statistical analyses and exemplary flow cytometry plots are given in **Supplementary File 4**). Taken together, these results indicate that bovine monocyte subsets are specialized in responding to different pathogen-associated molecular patterns, suggesting a role of ncM in immunity against viruses (TLR7/8) as well as gram-positive bacteria and mycoplasma (TLR2/6) (76) and for cM and intM against gram-negative bacteria (TLR4).

### Nonclassical monocytes have a gene expression signature promoting resolution of inflammation and tissue repair

**Figure 4:**
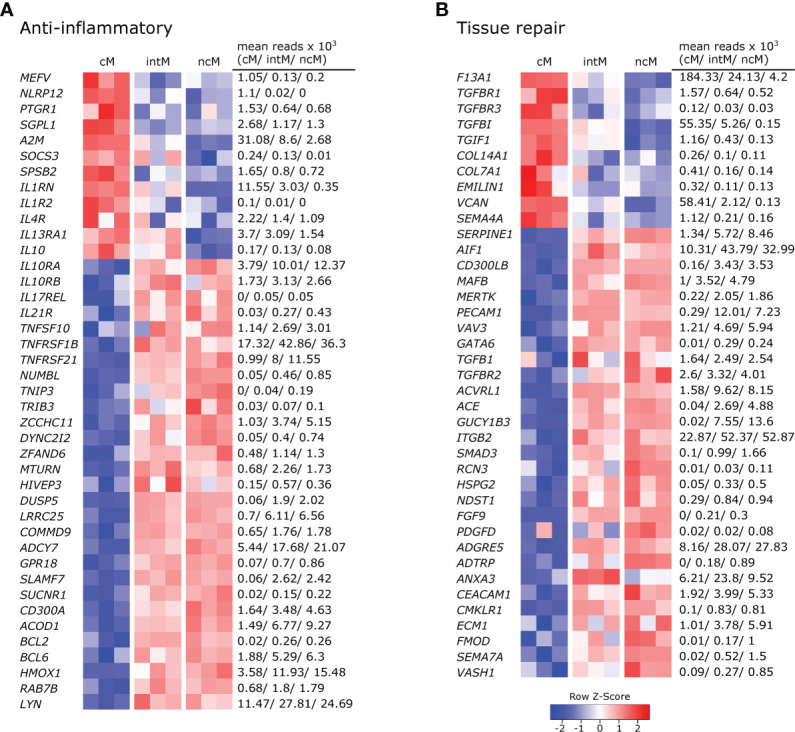
Gene expression associated with anti-inflammatory responses and tissue repair. Illumina sequencing was performed on RNA isolated from sorted monocyte subsets (cM, intM, ncM) of three animals. Heatmaps show row z-scores calculated from log2-transformed normalized counts of selected genes associated with anti-inflammatory responses **(A)**, and tissue repair **(B)**. Mean kilo reads for each subset and gene are given to the right of each heatmap. Genes were selected based on pairwise comparisons with DESeq2 (adjusted p-value < 0.05) and literature research.

All three monocyte subsets expressed anti-inflammatory genes, but ncM and intM were clearly dominant in this regard (**Figure 4A**).With the expression of anti-inflammatory genes, cM seemed to mainly regulate their own pro-inflammatory functions. Among those genes are regulators of inflammasome activation (*MEFV* (77), *NLRP12* (78)), an enzymatic inactivator of leukotriene B4 (*PTGR1*) and sphingosine-1-phosphate (*SGPL1*) (79), a cytokine-scavenging protein (*A2M*) (80), as well as a negative regulator of cytokine signaling (*SOCS3*) and nitric oxide production (*SPSB2*). Moreover, cM were strongly enriched in transcripts for the IL-1 receptor antagonist (*IL1RN*) and exclusively expressed the decoy receptor for IL-1 (IL1R2). Also *IL4R* and *IL13RA1* were expressed to higher levels in cM, suggesting that cM are especially receptive for IL-13, which was shown to inhibit the production of pro-inflammatory cytokines in macrophages (81). Furthermore, classical signaling through the IL-6 receptor (*IL6R*), 3-fold enriched in cM over ncM (**Figure 2A**), is reported to mediate anti-inflammatory effects of IL-6, as opposed to signaling through soluble IL-6 receptor (37). Notably, while cM expressed the highest levels of *IL10*, transcripts for the IL-10 receptor (*IL10RA*, *IL10RB*) were clearly enriched in ncM and intM. Additionally, both ncM and intM contained *IL17REL* transcripts, coding for a soluble receptor and potential negative regulator of IL-17 signaling (82). Moreover, ncM and intM were enriched for *IL21R* transcripts, with IL-21 signaling described to enhance SOCS gene expression and to limit cytokine production in human monocyte-derived cells (83). Also transcripts for TRAIL (*TNFSF10*), TNFR2 (*TNFRSF1B*) and DR6 (*TNFRSF21)* were strongly overexpressed in ncM and intM. Apoptosis-inducing TRAIL expression on monocytes is suggested to be critical for regulation of inflammation (84), and high expression of TNF receptor 2 (*TNFRSF1B*) in intM and ncM may favor suppressive signaling of transmembrane TNF-α, as reported for murine myeloid-derived suppressor cells (85). Death receptor 6 (*TNFRSF21*) has been reported to have inhibitory effects on monocyte differentiation when cleaved from the surface of tumor cells by matrix metalloproteinase 14 (*MMP14*) (86), the latter being expressed about 10-fold higher in ncM and intM (data not shown).

Other anti-inflammatory genes over-expressed by ncM and intM are reported to be mostly involved in negative regulation of NF-κB signaling (*NUMBL*, *TNIP3*, *TRIB3* (87), *ZCCHC11*, *DYNC2I2* (88), *ZFAND6* (89), *MTURN* (90), *HIVEP3* (91), *DUSP5*, *LRRC25*, *COMMD9* (92), *ADCY7* (93)), but also include surface receptors involved in regulation of inflammation, such as *GPR18* (receptor for resolvin D2) (94), *SLAMF7* (95), *SUCNR*1 (96), and *CD300A* (97).

Notably, expression of *ACOD*1 was over 6-fold higher in ncM compared to cM. The metabolite itaconate, generated by IRG1 (ACOD1), is well-known for its anti-inflammatory effects (66, 98). Furthermore, *BCL2* and *BCL6* were transcribed to higher levels in ncM and intM. BCL-2 was shown to negatively regulate caspase-1 activation (99) and BCL-6 was recently reported to exert anti-inflammatory effects by suppressing *IL6* transcription in murine macrophages (100). Like in human CD16^+^ monocytes (101), *HMOX1* was significantly increased in ncM and intM. Heme oxygenase-1 (HMOX1) was shown to be induced by IL-10 and to mediate the anti-inflammatory effect of IL-10 in murine macrophages, presumably *via* NF-κB suppression by the heme degradation product carbon monoxide (102). Also in human monocytes, HMOX1 was reported to inhibit LPS-induced TNF-α and IL1-β production (103). Additionally, *RAB7B*, described to promote degradation of TLR4 (104) and TLR9 (105), was more than 2-fold higher expressed in ncM and intM. As was *LYN*, also expressed in human ncM and intM (106, 107), and reported to negatively regulate TLR-induced cytokine responses (108). Notably, LYN has recently been proposed as a negative regulator of murine ncM development (109). Overall, these results indicate anti-inflammatory functions for ncM and intM.

In line with their suggested anti-inflammatory and pro-resolving functions, a number of genes associated with different stages of tissue repair were upregulated in ncM and intM (**Figure 4B**). While cM were enriched in *F13A1*, mediating hemostasis (110), intM and ncM expressed *SERPINE1*, described to regulate clot resolution (111). Both ncM and intM contained the highest transcript levels of genes associated with efferocytosis [*AIF1* (112), *CD300LB* (113), *MAFB* (114), *MERTK* (115), *PECAM1 (* 116), *VAV3* (117)]. Notably, MERTK has also been described as a negative regulator of human T-cell activation (118).

Furthermore, ncM expressed higher levels of *TGFB1* and of genes coding for TGF receptors (*TGFBR2*, *ACVRL1*) or being directly or indirectly involved in TGF-β pathways (*ACE*, *GUCY1B3*, *ITGB2*, *SMAD3*). Transforming growth factor beta (TGF-β) is known as a pro-fibrotic cytokine involved in wound healing (119). Notably, cM expressed higher levels of the TGF receptor genes *TGFBR1* and *TGFBR3*, and of *TGFBI* and *TGIF1*. Genes associated with extracellular matrix components dominantly transcribed by ncM included *HSPG2* (basement membrane-specific heparin sulfate) and *NDST1* (biosynthesis of heparin sulfate), whereas cM contained the highest transcript levels for collagens *COL14A1*, *COL7A1*, as well as for *EMILIN1* (associates with elastic fibers) and *VCAN* (versican). Furthermore, fibroblast growth factor *FGF9* and platelet-derived growth factor *PDGFD* were clearly enriched in ncM. The high expression of *CMLKR1* in intM and ncM suggests that these subsets are attracted to inflamed tissues *via* chemerin and contribute to resolution of inflammation *via* binding to resolvin E1 (120), which was shown to increase IL-10 production and phagocytosis of apoptotic neutrophils in macrophages (121, 122).

In addition, several metalloproteinases and their regulators were either upregulated in ncM (*ADAM9*, *ADAMTSL5*, *ADAMTS12*, *MMP14*, *MMP19*, *TIMP2*, *TSPAN14*, *ECM1*) or cM (*ADAM19*, *ADAM8*, *ADAMTS2*, *MMP25*) (data not shown). Several metalloproteinases are described to be involved in wound healing (123), among which MMP14 and ADAM9 are suggested to regulate epithelial cell proliferation (123, 124). Finally, several genes associated with angiogenesis (*ADGRE5*/CD97 (125), *ADTRP*, *ANXA3* (126), *CEACAM1* (127), *CMKLR1* (128), *ECM1* (129)*, FMOD* (130), *SEMA7A* (131), *VASH1*) were predominantly expressed by intM and ncM. Notably, *SEMA4A* (132), also reported to be involved in angiogenesis, showed the highest transcription in cM. Altogether, these data suggest anti-inflammatory pro-resolving functions of ncM and intM, as well as a prominent role of these subsets in wound healing and tissue regeneration.

### Gene expression indicates differential capabilities for antigen presentation, co-stimulation, and modulation of T-cell responses

**Figure 5:**
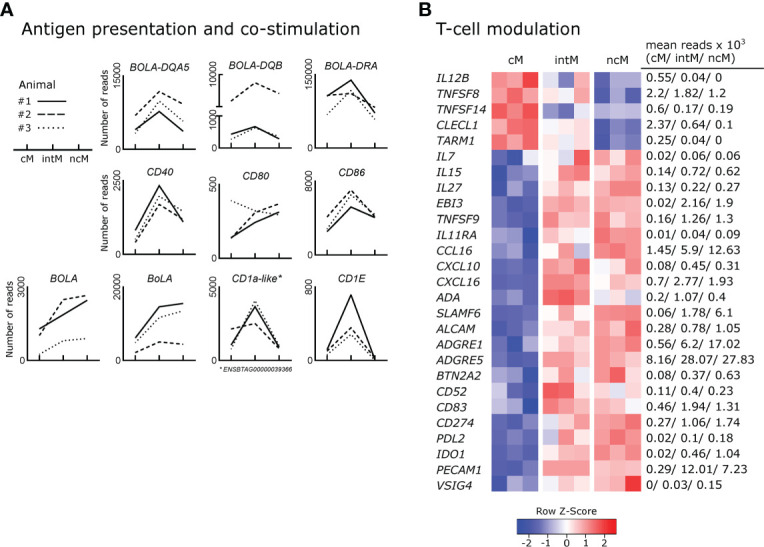
Expression of genes involved in the shaping of T-cell responses. Illumina sequencing was performed on RNA isolated from sorted monocyte subsets (cM, intM, ncM) of three animals (#1-3). **(A)** Gene expression promoting antigen presentation and co-stimulation. Graphs show the number of reads across monocyte subsets for selected genes with individual animals indicated by solid (#1), dashed (#2) and dotted (#3) lines. **(B)** Gene expression involved in T-cell modulation. Heatmap shows row z-scores calculated from log2-transformed normalized counts of selected genes. Mean kilo reads for each subset and gene are given to the right of the heatmap. Genes were selected based on pairwise comparisons with DESeq2 (adjusted p-value < 0.05) and literature research.

Antigen presentation capabilities are reported for monocytes across species (1). As expected from phenotypic analyses, intM stood out by their high gene expression for MHC-II (*BOLA-DQA5*, *BOLA-DQB*, *BOLA-DRA*), and the co-stimulatory molecules CD40 and CD86 (**Figure 5A**). Interaction of CD40 with CD40L on T cells has been reported to stimulate Th17 responses (133). Notably, genes for MHC class I molecules (BOLA, BoLA), were transcribed to higher levels in intM and ncM. Furthermore, mRNA from genes associated with the presentation of lipid antigens to T cells (ENSBTAG00000039366 annotated as CD1a molecule-like, *CD1E*), was enriched in intM.

Genes encoding T-cell signaling cytokines predominantly expressed by cM included *IL12B* (**Figure 5B**), as well as *TNFSF8* (CD30L) and *TNFSF14* (LIGHT) (134, 135). In fact, transcription of *IL12B* mRNA was found to be absent in ncM, and over 13-fold increased in cM over intM. Two further genes upregulated in cM and reported to be involved in regulation of T-cell activation include *CLECL1* and *TARM1*. *CLECL1*, 20-fold increased over ncM, has been reported to act as a T-cell costimulatory molecule, skewing the CD4 T-cell response towards Th2 by increasing IL-4 production and proliferation (136). *TARM1*, about 6-fold enriched in cM, has been reported to suppress CD4-T-cell activation and proliferation *in vitro* (137).

T-cell signaling cytokines predominantly expressed by intM and ncM included *IL7* and *IL15*, reported to function in lymphoid homeostasis (138), and *IL27*, encoding a multifaceted cytokine described to both promote and suppress T-cell responses (139). Also, *EBI3* (IL-27β), an essential component of the cytokines IL-27 and IL-35, was exclusively expressed in intM and ncM (approx. 110-fold increased). Moreover, *TNFSF9*, a ligand for CD137 on T cells shown to be important for the generation of antiviral CD8-T-cell responses (140), was 8-fold enriched in ncM over cM.

Furthermore, *IL11RA* (alpha subunit of the IL-11 receptor) was expressed at higher levels in ncM and intM. In line with almost absent *IL12B* transcription in ncM and intM, IL-11 signaling has been reported to inhibit IL-12 production in macrophages (141), which supports polarization towards Th2 responses (142). As shown in **Figures 2B**, **5B**, chemokines relevant for T-cell responses were mainly expressed by ncM and intM (*CCL3*, *CCL4*, *CCL5* and *CCL16*, *CXCL10*, *CXCL16*).

In fact, the vast majority of genes (22 out of 27) associated with modulation of T-cell responses was overexpressed in ncM and intM (**Figure 5B**). Intermediate monocytes expressed the highest levels of adenosine deaminase (*ADA*), which is reported to act as a modulator of T-cell differentiation, increasing the generation of effector, memory, and regulatory T cells (143). Both ncM and intM were enriched in transcripts for *SLAMF6*, reported to boost IFN-γ production and cytolytic anti-tumor activity of human CD8 T cells *in vitro* (144). Furthermore, expression of *ALCAM*, encoding a ligand for CD6 on T cells, important for stabilizing the immunological synapse between APC and T cells (145) and reported to mediate extravasation of monocytes (146), was more than 3-fold higher expressed in ncM than in cM. Notably *ADGRE1* (F4/80), reported to be essential for the generation of Tregs and peripheral tolerance when expressed on antigen-presenting cells (147), was 30-fold enriched in ncM over cM. Moreover, *ADGRE5*, coding for CD97 and described to induce regulatory T cells and IL-10 production upon engagement of CD55 on CD4 T cells (148, 149) was 3-fold higher transcribed in ncM and intM. Consistent with the idea that ncM promote the generation of Tregs, they showed the highest transcription of *BTN2A2*, a butyrophilin reported to inhibit activation and induce Foxp3 expression in murine T cells (150, 151).

Many T-cell modulating genes overexpressed in ncM and intM were found to be genes involved in negative regulation of T-cell activation (*BTN2A2*, *CD52*, *CD83*, *CD274*/PDL1, *PDL*2, *IDO1*, *PECAM1*, and *VSIG4*). Soluble CD52 was reported to suppress T-cell activation *via* binding to Siglec-10 (152) and soluble CD83 was shown to regulate T-cell activation by binding to the TLR4/MD-2 complex on human CD14^+^ monocytes and inducing expression of anti-inflammatory mediators such as IDO and IL-10 (153). Transcription for PDL-1 *(CD274)*, a well-known inhibitor of T-cell activation (154), was increased 6-fold in ncM over cM. Notably, PDL-1 has recently been employed as a marker of ncM for *in-vivo* tracking of this monocyte subset in mice (155). Similarly, the gene for PDL-2, a second ligand for PD-1 on T cells with T-cell inhibitory function (156), was 9-fold higher expressed in ncM, though at lower levels than the gene for PDL-1 (CD274). Strikingly, *IDO1* expression was found to be significantly increased in intM (20-fold) and ncM (50-fold) when compared to cM, where expression was almost absent (mean of 20 reads). IDO1 was described to inhibit T-cell activation by degrading tryptophan, and to promote tolerance of DC and the expansion of Tregs (157). Also transcription of *PECAM1* (CD31), described as a key co-inhibitory receptor promoting tolerogenic functions in both DCs and T cells through homophilic interactions (158), was greatly upregulated in ncM and intM. A recent *in vitro* study also suggested that high CD31 expression on DCs reduces priming of CD4 T cells by impairing stable cell-cell contacts (159). Furthermore, *VSIG4*, coding for a B7-family related protein specifically expressed on resting macrophages was primarily expressed in ncM. Notably, VSIG4 has been described as a strong negative regulator of T-cell activation, maintaining T-cell unresponsiveness in healthy tissues (160). Taken together, these data clearly indicate that monocyte subsets are actively involved in the shaping of T-cell responses, with ncM and intM being especially well equipped for T-cell suppression, either directly or *via* the induction of regulatory T cells.

### Expression of metabolic genes differs markedly between classical and nonclassical monocytes

**Figure 6:**
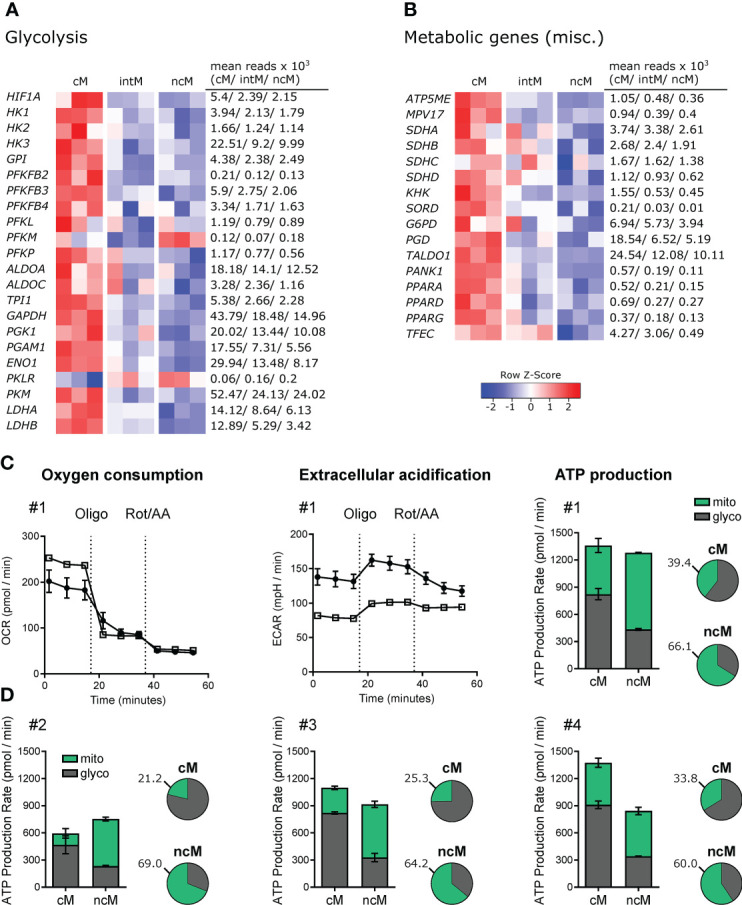
Metabolic gene expression and Agilent Seahorse Assays. **(A, B)** Illumina sequencing was performed on RNA isolated from sorted monocyte subsets (cM, intM, ncM) of three animals. Heatmaps show row z-scores calculated from log2-transformed normalized counts of glycolytic genes **(A)** and of genes associated with other metabolic pathways **(B)**. Mean kilo reads for each subset and gene are given to the right of each heatmap. Genes were selected based on pairwise comparisons with DESeq2 (adjusted p-value < 0.05) and literature research. (C+D) Bovine monocyte subsets were FACS-sorted and metabolic activity was analyzed by Agilent Seahorse XF technology and the XF Real-Time ATP Rate Assay. Oxygen consumption rate (OCR) and extracellular acidification rate (ECAR) were automatically calculated from measured oxygen and pH decrease (Agilent Wave software). The electron transport chain inhibitors oligomycin and rotenone/antimycin A were injected sequentially to allow calculation of OXPHOS- as well as glycolysis-mediated ATP production rates (mito, glyco) from resulting OCR and ECAR. Monocyte subsets from four animals were analyzed. **(C)** For one representative animal, OCR and ECAR traces are depicted in addition to ATP production rates which are shown as absolute values (bar graphs) and relative values (pie charts). **(D)** ATP production rates for three other animals. Data of intM (2 animals) and monocyte-derived macrophages (2 out of 3 animals) is shown in **Supplementary File 6**.

Given that metabolism and immune function are tightly linked (161–163), differences in metabolic pathways can give indications for subset-specific functions. In fact, monocyte subsets showed prominent differences in the expression of genes relating to metabolism. The vast majority of these differentially expressed genes was associated with glycolysis and showed the highest expression in cM (**Figure 6A**). Apart from glycolytic genes, also genes involved in oxidative phosphorylation showed increased transcription in cM as compared to ncM (**Figure 6B**). Among those genes was *ATP5ME* (subunit of the mitochondrial ATP synthase), a mitochondrial inner membrane protein (*MPV17*) supporting oxidative phosphorylation (164), the genes coding for the components of complex II of the mitochondrial electron transport chain succinate dehydrogenase (*SDHA*, *SDHB*, *SDHC*, *SDHD*), and also most of the numerous genes coding for subunits of complex I (data not shown). In line with increased glycolysis in cM, transcripts for the glucose transporters *SLC2A1* and *SLC2A3* were about 3-fold enriched in cM (data not shown). As reported in our previous publication (9), also SLC genes involved in transport of succinate (*SLC13A3*), citrate (*SLC13A5*) and lactate/pyruvate (*SLC16A1*) were mainly expressed in cM. Notably, the metabolites succinate and citrate are both described as critical pro-inflammatory mediators linking metabolism to immune functions (165). Moreover, two genes associated with fructose metabolism (*KHK*, *SORD*) were predominantly expressed in cM. Fructose-induced metabolic changes were recently described to enhance inflammatory responses of dendritic cells (166). In addition, genes involved in the oxidative (*G6PD*, *PGD*) and non-oxidative (*TALDO1*) pentose-phosphate pathway (PPP) showed the highest transcription in cM. The PPP, providing redox-equivalents and nucleotide precursors, was shown to be essential for pro-inflammatory functions of human macrophages (167, 168). Furthermore, *PANK1*, coding for a key enzyme in CoA synthesis was 5-fold upregulated in cM over ncM.

Notably, all three genes coding for members of the PPAR family (*PPARA*, *PPARD*, *PPARG*) were transcribed at higher levels in cM. These lipid-activated nuclear receptors have evolved as key regulators linking lipid metabolism to inflammation, and in particular expression of PPARG is associated with anti-inflammatory functions (169). We recently showed that bovine cM downregulate *PPARG* transcription dramatically upon Gardiquimod (TLR7/8) stimulation *in vitro* (24). PPARA was recently proposed as an important mediator of antimicrobial responses to mycobacteria (170), inducing expression and translocation of TFEB, a key transcriptional activator of autophagy and lysosomal biogenesis. Notably, while *TFEB* was equally expressed in all monocyte subsets (data not shown), transcription of *TFEC*, a less well described member of the TFE family, was approximately 9- and 6-fold increased in cM and intM respectively compared to ncM.

Altogether, these data suggest that cM are metabolically more active than ncM and intM, with a significantly enhanced expression of genes involved in glycolysis, supporting pro-inflammatory functions.

### Mitochondrial respiration prevails in nonclassical monocytes

To investigate differential use of metabolic pathways in monocyte subsets, we assessed the contribution of glycolysis and mitochondrial respiration to their ATP production. Extracellular flux analysis of sorted monocyte subsets demonstrated that cM produced the majority of their ATP through glycolysis, whereas ncM predominantly used oxidative phosphorylation (OXPHOS) for ATP production (**Figure 6C, D**). Raw data and statistical analyses (ATP production rates) are shown in **Supplementary File 5**.

Intermediate monocytes appeared to proportionally produce more ATP *via* glycolysis than ncM (**Supplementary File 6**), however these results should be interpreted with caution, as bulk RNA-seq of intM revealed pronounced animal-to-animal variability, presumably caused by considerable heterogeneity, or mixed populations within the intM gate.

Monocyte-derived macrophages (cM-M, intM-M, ncM-M) generated by a 6-day *in-vitro* culture showed considerably higher metabolic activity evident also by the need to reduce the cell number for Seahorse assays by five times. Notably, after this 6-day culture, the proportion of ATP produced by OXPHOS was increased in all subsets (**Supplementary Files 6C, D**; right panels). It remains to be elucidated whether this switch to OXPHOS is a hallmark of macrophage differentiation or rather a result of *in-vitro* culture conditions. Certainly, the observed preferences for different metabolic pathways *ex vivo* are in line with diverging roles of monocyte subsets in inflammation and beyond.

### Gene set enrichment analysis

In addition to the manual gene-by-gene analysis described above, automated analyses with pre-defined gene sets were performed for the bulk RNA-seq datasets. Intermediate monocytes were excluded from the analyses due to the high animal-to-animal variability observed for certain genes. Enrichment analysis with gene ontology gene sets related to biological processes was found to be most informative. In line with the manual analysis, genes overexpressed in cM vs. ncM (DESeq2 output; adjusted p-value < 0.05) were enriched (q value < 0.05) in gene sets related to metabolism as well as to antibacterial and pro-inflammatory responses, whereas genes overexpressed in ncM vs. cM were enriched in gene sets related to adhesion, T-cell regulation, wound healing and antiviral responses.

The complete list of gene sets is provided as **Supplementary File 7**, including the lists of genes assigned to these gene sets, which should be carefully examined before making further conclusions. Especially for genes overexpressed in ncM, many gene sets were found to be misleading, suggesting for example an involvement in B-cell responses due to genes expressed in, and related to, B cells.

### Single-cell transcriptomic data suggests continuous differentiation

**Figure 7:**
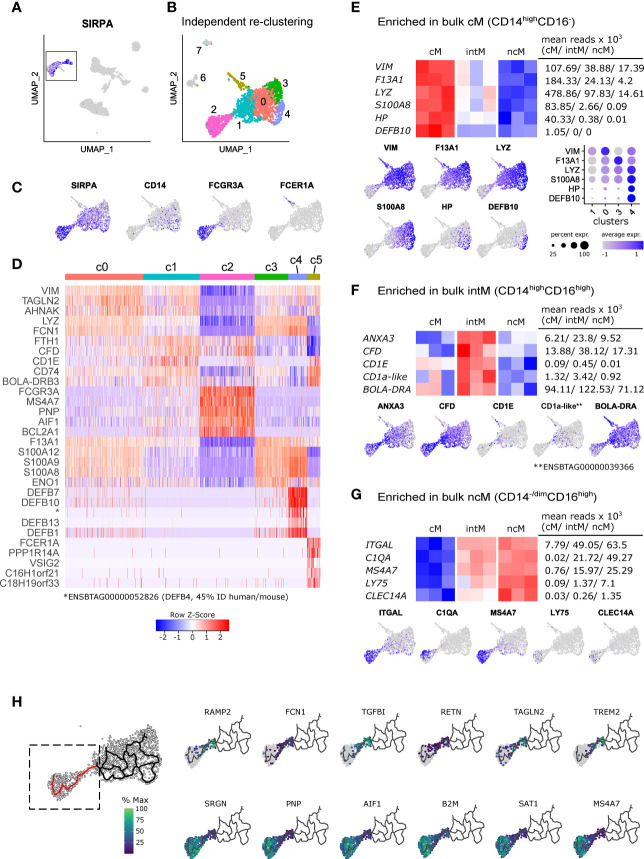
Single-cell RNA sequencing reveals heterogeneity of bovine monocytes. PBMC from two cows were subjected to 10x Genomics 3’ single-cell RNA sequencing. **(A, B)** Clusters containing *SIRPA*-expressing putative monocytes were subsetted for independent re-clustering. Clusters 6 and 7 were excluded from further analysis. **(C)** Feature plots show expression of *SIRPA*, *CD14*, *FCGR3A* (CD16) and *FCER1A* in the re-clustered dataset. **(D)** Heatmap shows the top 5 (adjusted p-value) differentially expressed genes, revealing expression signatures resembling classical monocytes (c0, c3, c4), intermediate monocytes (c1, c2), nonclassical monocytes (c2), and cDC2 (c5). Differential expression testing was performed with seurat’s FindAllMarkers function. **(E-G)** Visualization of selected signature genes enriched in bulk-sequenced cM **(E)**, intM **(F)**, and ncM **(G)**. **(H)** Trajectory analysis on clusters 0-4 and visualization of genes differentially expressed along a selected trajectory (indicated in red). The complete list of differentially expressed genes along this trajectory is given in **Supplementary File 11**.

Single-cell RNA-seq has provided unprecedented insights into the heterogeneity of myeloid cells (171), highlighting that the classification of human and bovine monocyte subsets according to expression of CD14 and CD16 may be an oversimplification. In order to get an unbiased view on monocyte subset composition, we have performed 10x Genomics single-cell RNA-seq of bovine PBMC (summaries of Cell Ranger outputs are given in **Supplementary File 8**). Within PBMC, monocytes were readily identified by visualizing *SIRPA* expression in the UMAP projection (**Figure 7A**). To better resolve potential subclustering, clusters belonging to this group of *SIRPA*-expressing cells (c4, c10, c14, c18; **Supplementary File 9A**) were selected and re-clustered independently from the other cells in the PBMC dataset (**Figure 7B**). From the resulting eight clusters (resolution 0.6), only six clusters (clusters 0-5) were further analyzed, as clusters 6 and 7 appeared to contain doublets with expression of T- and B-cell associated genes alongside *SIRPA* expression (**Supplementary File 9B**). Visualization of key genes and analysis of the top differentially expressed genes identified cluster 2 as ncM/intM and cluster 5 as cDC2 (**Figures 7C, D**). Clusters 0, 3 and 4 appeared to contain cM in different activation states. Notably, cluster 4 stood out by high expression of several defensin genes. The complete list of differentially expressed genes is given in **Supplementary File 10**.

Visualization of signature genes derived from bulk-sequenced cM supported their cluster annotation, and for some genes (e.g. *LYZ*, *S100A8*), also revealed a gradual expression increase from clusters 1 and 0 towards clusters 3 and 4 (**Figure 7E**), while *VIM* and *F13A1* showed the highest transcription in cluster 0 and 3, respectively. In line with their intermediate nature, transcripts enriched in bulk-sequenced intM (e.g. *ANXA3*, *CFD, CD1E*) were mostly detected in cells connecting clusters 1 and 2 (**Figures 7B, F**). It must be noted that bulk sequenced intM of the present study were defined as CD14^high^CD16^high^. It is expected that cluster 1 contains CD14^high^CD16^dim^ cells, which were not included in the sorting gate used for bulk RNA sequencing. Transcripts enriched in bulk-sequenced ncM were almost exclusively detected in cluster 2 (**Figures 7B, G**). Notably, expression of *C1QA* (**Figure 7G**), as well as of *C1QB* and *C1QC* (not shown) appeared to be restricted to a subcluster within cluster 2, suggesting the presence of distinct cell states within ncM.

Trajectory analyses performed with Monocle 3, resulted in net-shaped trajectories spanning clusters 0, 1, 3 and 4, and a straight trajectory from cluster 1 towards cluster 2. Genes for which expression either significantly (q value < 0.05) decreased or increased along this straight trajectory are shown in **Figure 7H** (selection) and **Supplementary File 11** (complete list).

**Figure 8:**
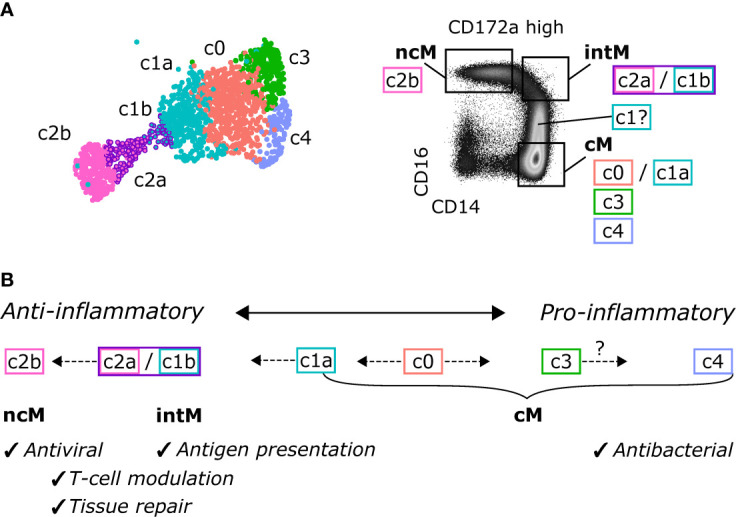
Proposed continuous differentiation and proposed functional specialization of bovine monocyte subsets. **(A)** Assignment of scRNA-seq clusters to CD14/CD16-defined monocyte subsets, as sorted for bulk RNA-seq. **(B)** Proposed differentiation pathways. A subcluster of cM (c0) may give rise to anti-inflammatory intM and ncM with specialized functions in antigen presentation and antiviral responses, respectively, and to pro-inflammatory subclusters of cM with prominent antibacterial functions.

Taken together, the unbiased analysis of single-cell transcripts revealed remarkable heterogeneity of bovine blood-derived cM in healthy animals, presumably reflecting a continuum of activation states, and supports the hypothesis that ncM are generated *via* differentiation from cM, with intM (CD14^high^CD16^high^), or a subpopulation thereof, representing a transient intermediate state, potentially specialized in lipid antigen presentation to T cells (**Figure 8**). Future studies need to address CD14^high^CD16^dim^ monocytes and the heterogeneity of CD14^high^CD16^high^ intermediate monocytes.

## Discussion

With the present study, we extended the phenotypic characterization of bovine monocyte subsets and combined an in-depth analysis of their bulk- and single-cell transcriptomes with metabolic and TLR-stimulation assays to get detailed insights into subset-specific functions. Pairwise comparison of bulk gene expression coupled with extensive literature research revealed substantial transcriptomic differences between bovine monocyte subsets, likely determining their specializations, while single-cell transcriptomics provided an unbiased view on subset composition supporting differentiation of cM towards ncM *via* relatively transient intM. Genes differentially expressed between bulk-sequenced cM and ncM were also analyzed by GSEA with GO_BP gene sets. While GSEA largely confirmed the manual analysis, results of GSEA need to be interpreted with caution and require manual validation with respect to gene-set composition.

Bovine cM clearly emerged as pro-inflammatory, with overall gene expression supporting antibacterial inflammatory responses. This is in line with data on human and murine cM, and with earlier studies on bovine cM that have shown their superiority in phagocytosing bacteria (34). Both ncM and intM were dominant in the transcription of many genes associated with regulatory functions. The expression of anti-inflammatory genes and numerous genes associated with wound healing (efferocytosis, angiogenesis, fibrosis) clearly indicate that bovine ncM are specialized in the resolution of inflammation and in tissue regeneration, as suggested for ncM based on studies in mouse models (172). In line with our steady-state transcriptomic data, bovine ncM were previously shown to almost lack IL-1β production upon inflammasome activation *in vitro* (34). However, literature on the ability of human ncM to produce IL1-β is conflicting (6, 173).

Pro- and anti-inflammatory functions of cM and ncM, respectively, are also supported by their metabolic transcriptome, clearly indicating that cM are metabolically more active and skewed towards pro-inflammatory glycolysis. Differential use of ATP-generating metabolic pathways could also be confirmed by extracellular flux analysis, where cM predominantly performed glycolysis and ncM mainly employed oxidative phosphorylation (OXPHOS). These metabolic differences are in line with reported lower glucose uptake of bovine ncM as compared to cM (174), and match gene expression as well as respirometric measurements in human cells (175), suggesting similar metabolic programing and functional specialization of monocyte subsets in humans and cattle.

Transcriptomic data also suggest diverging functions of bovine monocyte subsets in the interaction with T cells. Notably, intM showed the highest expression of MHC-II, both on mRNA and protein level. High expression of MHC-II is also reported for human intM (5, 6), and may be linked to their superiority in stimulating human CD4-T-cell proliferation (5). Also considering the high expression of CD86 and the high transcription of *CD1E* and a CD1a-like gene, bovine intM may be particularly well equipped for co-stimulation, and lipid antigen presentation to T cells. A specialization in antigen presentation is also reported for human intM (15). Nonclassical monocytes and intM were enriched in transcripts for various genes promoting CD8-T-cell responses. This preferential activation of CD8 T cells and the risk associated with uncontrolled cytolytic T-cell responses might explain why ncM and intM also show high expression of genes mediating the inhibition of T cells and the generation of Tregs, the latter being reported for murine ncM (176). Furthermore, gene expression promoting activation of CD8 T cells, together with the interferon-associated gene signature and the high responsiveness to Gardiquimod and Resiquimod, indicate a specialization of bovine ncM towards antiviral responses, as suggested for human ncM (14).

In support of T-cell stimulating functions, we have detected monocytic cells transcriptionally resembling ncM, intM and cM in bovine mesenteric lymph nodes (scRNA-seq; manuscript in preparation), however the mechanisms of lymph-node entry remain elusive. In contrast to ncM and intM, which almost lack CD62L expression, bovine CD62L^high^ cM should be able to enter lymph nodes directly from blood. Also lymph-mediated entry of antigen-presenting monocytes has been reported for mice (177), however we could not observe CCR7 upregulation in/on stimulated bovine monocytes (24), making lymph-mediated entry *via* CCR7 rather unlikely.

As reported for human intM (6), bovine intM expressed the majority of genes at levels in-between cM and ncM, while showing higher transcriptional similarity with ncM. Notably, bovine intM were reported to produce the highest amounts of reactive oxygen species in response to opsonized bacteria and the highest amounts of IL-1β following inflammasome activation (34). This is surprising when looking at the steady-state transcriptome of intM described in the present study. A major limitation of studies on intM across species is their poor phenotypic definition, likely including multiple subsets or different activation states leading to conflicting results (11). In fact, heterogeneity of intM has been described for both humans (171) and mice (178). The lack of a clear cluster assignment in our scRNA-seq data and the observation that animal-to-animal variability was most prominent for intM in the bulk dataset support the idea that also bovine CD14^high^CD16^high^ intM are a mixed population – or at least contain various cell states. Notably, a reported expansion of intM in response to dengue-virus infection was recently revealed to be an upregulation of CD16 on human cM (179). Also for bovine cM, an upregulation of CD16 is reported following stimulation with IFN-γ (34). Similarly, we found that sorted bovine cM (CD14^+^CD16^-^) were all CD16^high^ after overnight culture (unpublished observation), making them phenotypically indistinguishable from intM when using the standard gating strategy with CD14 and CD16. Therefore, also dominant glycolysis and LPS responsiveness of intM, observed in the present study and both reminiscent of cM, should be interpreted in the light of possible gate contamination with cM that have recently upregulated CD16 expression – potentially a phenotypic alteration that precedes profound transcriptomic alterations in intM.

It is widely accepted that murine and human intM and ncM arise from cM in the periphery (19, 180, 181). Our single-cell RNA-seq data enable an unbiased view on monocyte subset composition in bovine blood and support the idea of sequential differentiation. Along this line, intM – as identified by bulk-derived signature genes – appeared to be bridging between cM and ncM in the UMAP projection of our single-cell dataset. Further studies are required to understand the differentiation trajectories and to elucidate whether bovine ncM can differentiate from cM under steady-state and/or inflammatory conditions and if and how they can enter tissues to fulfill their specialized functions during immune responses induced by infections and vaccinations. Given the striking similarities of bovine and human monocyte subsets (15), insights from studies in cattle should also advance our understanding of monocyte biology and associated diseases in humans.

## Data availability statement

The original contributions presented in the study are publicly available. This data can be found here: [https://www.ebi.ac.uk/ena/browser/home/PRJEB50632].

## Ethics statement

The animal study was reviewed and approved by the committee on animal experiments of the canton of Bern, Switzerland, and the cantonal veterinary authority (Amt für Landwirtschaft und Natur LANAT, Veterinärdienst VeD, Bern, Switzerland) under the license numbers BE102/15, BE104/17, and BE131/17.

## Author contributions

ST and AS designed the study. ST performed laboratory work, analyzed and interpreted the data and wrote the manuscript. GTB performed laboratory work and analyzed flow cytometry data. HL and RB bioinformatically processed scRNA-seq data. RR and LvM assisted with Seahorse experiments and data interpretation. AS assisted with data interpretation and data analysis. All authors contributed to the article and approved the submitted version.

## Funding

Open access funding was provided by the University Of Bern.

## Acknowledgments

We want to thank the team of the Clinic for Ruminants at the Vetsuisse Faculty in Bern and the team of animal caretakers at the IVI in Mittelhäusern for blood sampling. Many thanks also go to Giuseppe Bertoni and Nicolas Ruggli for obtaining the cantonal licenses for blood sampling. We also want to thank Sylvie Python and Gael Auray for their support with cell sorting and RNA extraction and Corinne Hug for isolating PBMC and preparing monoclonal antibodies. Furthermore, we want to thank Stefan Müller and Thomas Schaffer (Flow Cytometry and Cell Sorting Facility, University of Bern) for cell sorting. Finally, we want to thank Pamela Nicholson and Tosso Leeb from the Next Generation Sequencing Platform of the University of Bern, and Irene Keller (Interfaculty Bioinformatics Unit, University of Bern) for their support with RNA-sequencing and bioinformatic analyses. A previous version of the manuscript has been uploaded to the preprint server bioRxiv (35).

## Conflict of interest

Author LvM is employed by Bucher Biotec AG.

The remaining authors declare that the research was conducted in the absence of any commercial or financial relationships that could be construed as a potential conflict of interest.

## Publisher’s note

All claims expressed in this article are solely those of the authors and do not necessarily represent those of their affiliated organizations, or those of the publisher, the editors and the reviewers. Any product that may be evaluated in this article, or claim that may be made by its manufacturer, is not guaranteed or endorsed by the publisher.
