# Erratum: Monocyte biology conserved across species: Functional insights from cattle

**DOI:** 10.3389/fimmu.2023.1180677

**Published:** 2023-03-15

**Authors:** Frontiers Production Office

**Affiliations:** Frontiers Media SA, Lausanne, Switzerland

**Keywords:** bovine monocyte subsets, classical monocytes, intermediate monocytes, nonclassical monocytes, transcriptome, single-cell RNA sequencing (scRNA-seq), immunometabolism, cattle

An omission to the funding section of the original article was made in error. The following sentence has been added: “Open access funding was provided by the University Of Bern”.

The original version of this article has been updated.

