# Supplementary material for: Monocyte biology conserved across species: Functional insights from cattle
Source: Front Immunol. 2022 Jul 29;13:889175. doi: 10.3389/fimmu.2022.889175 (PMC9373011; doi:10.3389/fimmu.2022.889175)
Supplement: Supplementary file 1 [file DataSheet_1.pdf]

Supplementary File 1

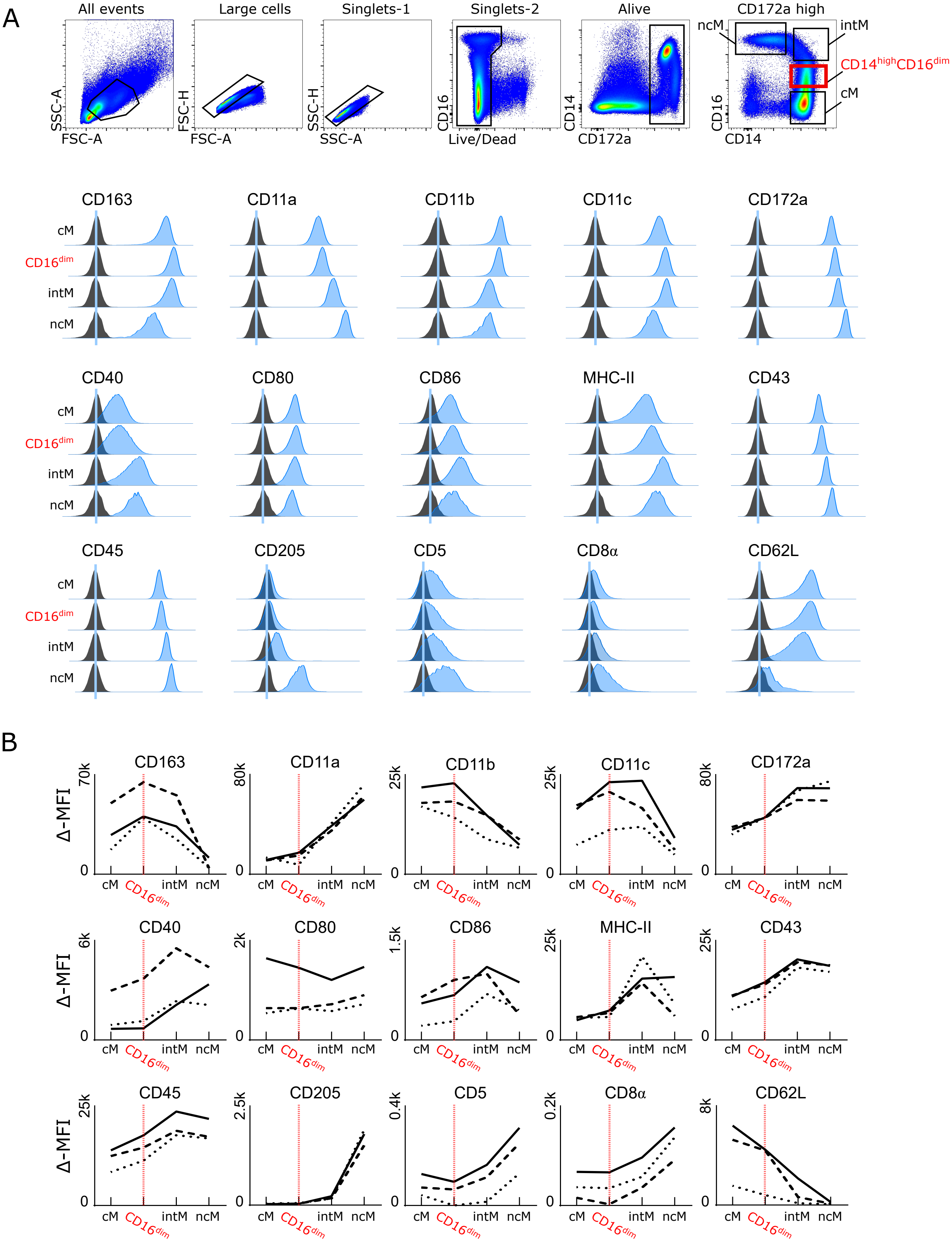

Supplementary File 1 Phenotype of bovine monocyte subsets, including CD14^{high}CD16^{dim} monocytes. **(A+B)** Flow cytometric analysis of freshly isolated PBMC **(A)** Gating strategy and exemplary flow cytometry data (corresponding to solid black line in B). Grey histograms represent aligned FMO controls. **(B)** Graphs show the delta median fluorescence intensity (MFI) of surface expression for selected molecules. Delta MFI was calculated as the difference in MFI between stained samples and FMO controls. Stainings were performed on seven different animals, resulting in 3 animals analyzed per marker. Within single graphs, data of three different animals is illustrated by solid, dashed, and dotted black lines. The red lines indicate CD14^{high}CD16^{dim} monocytes.
