# Supplementary material for: Monocyte biology conserved across species: Functional insights from cattle
Source: Front Immunol. 2022 Jul 29;13:889175. doi: 10.3389/fimmu.2022.889175 (PMC9373011; doi:10.3389/fimmu.2022.889175)
Supplement: Supplementary file 2 [file DataSheet_2.pdf]

Supplementary File 2

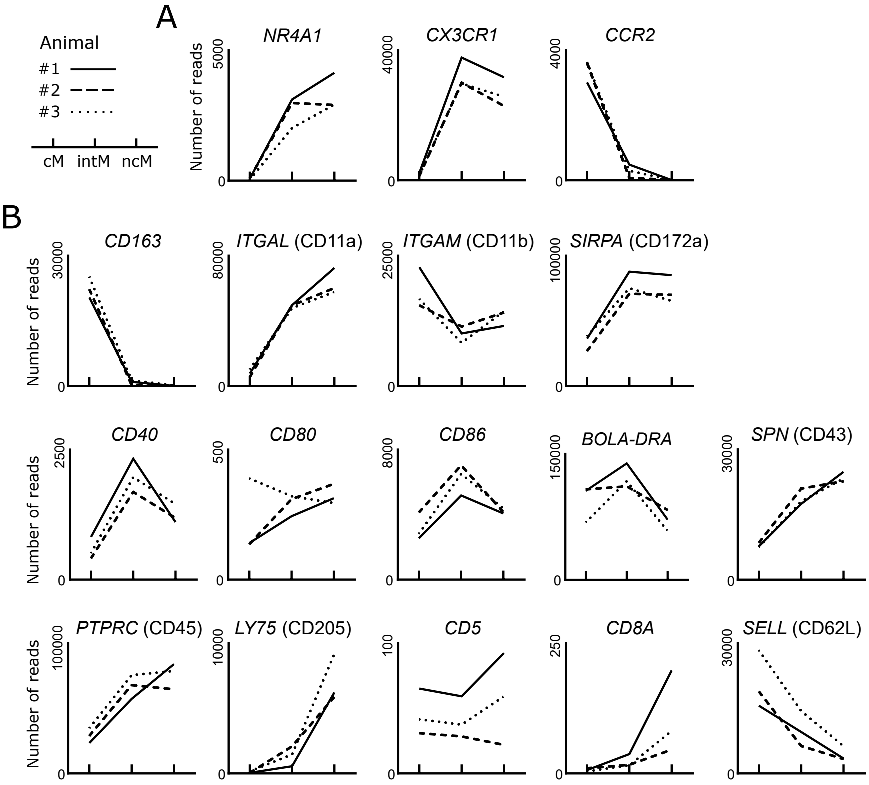

Supplementary File 2 Transcription of key genes **(A)** and genes previously analyzed by flow cytometry **(B)**. Illumina sequencing was performed on RNA isolated from sorted monocyte subsets (cM, intM, ncM) of three animals (#1-3). Graphs show the number of reads across monocyte subsets for selected genes with individual animals indicated by solid (#1), dashed (#2), and dotted (#3) lines.
