# Supplementary material for: Monocyte biology conserved across species: Functional insights from cattle
Source: Front Immunol. 2022 Jul 29;13:889175. doi: 10.3389/fimmu.2022.889175 (PMC9373011; doi:10.3389/fimmu.2022.889175)
Supplement: Supplementary file 4 [file DataSheet_4.pdf]

Supplementary File 4

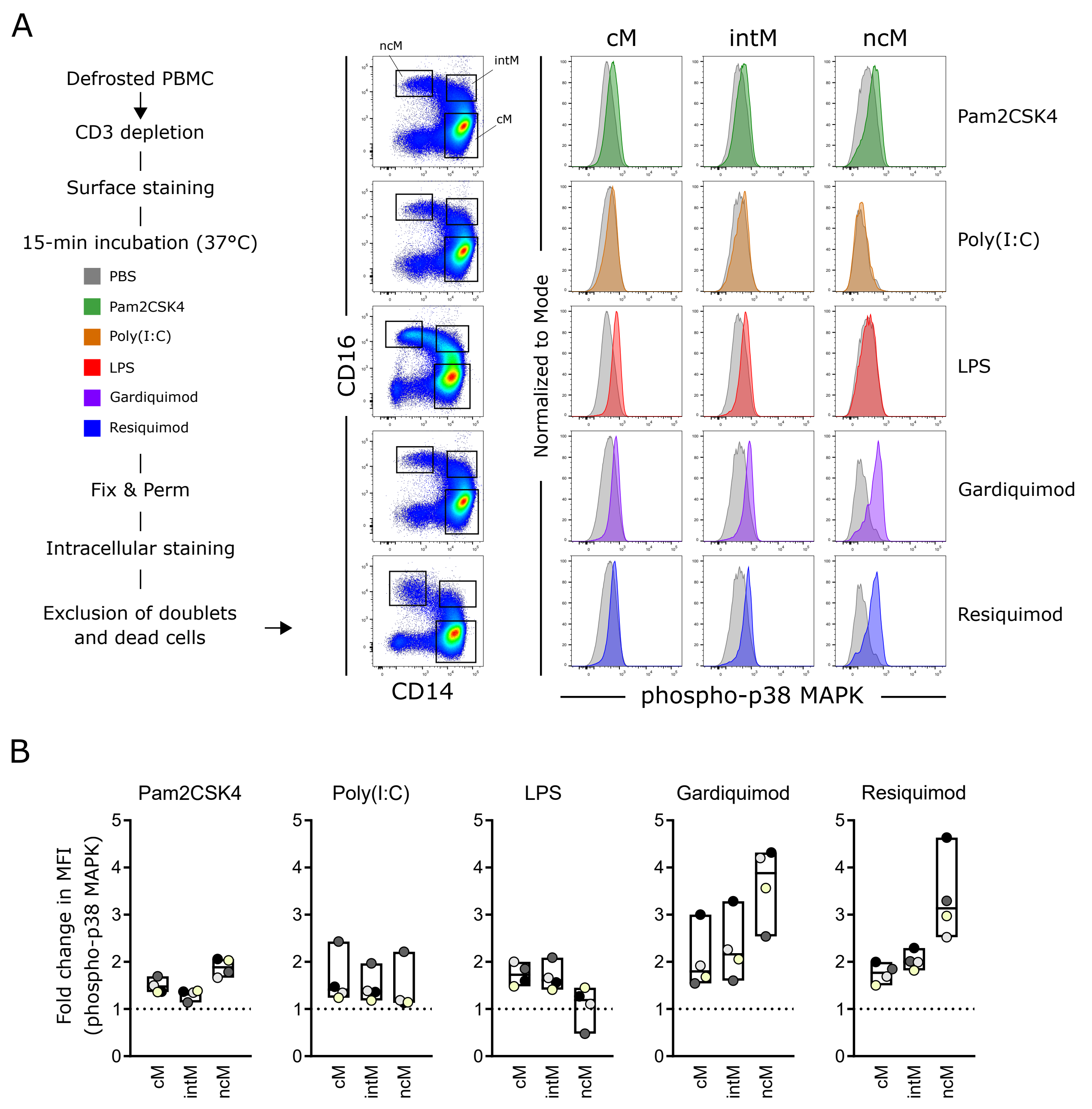

C

	p value	q value	adj. p value
Pam_cM	0.004799	0.002879	0.042368
Pam_intM	0.010242	0.00478	0.06953
Pam_ncM	0.001189	0.00236	0.016521
Poly_cM	0.059743	0.019301	0.168734
Poly_intM	0.042916	0.01502	0.160925
Poly_ncM	0.158402	0.047521	0.291712
LPS_cM	0.004179	0.002879	0.04101
LPS_intM	0.008732	0.004584	0.067761
LPS_ncM	0.981014	0.274684	0.981014
Gardi_cM	0.019728	0.007532	0.094824
Gardi_intM	0.01259	0.005288	0.073203
Gardi_ncM	0.001872	0.00236	0.024061
Resi_cM	0.00281	0.00236	0.031722
Resi_intM	0.000687	0.00236	0.010249
Resi_ncM	0.002683	0.00236	0.031722

Supplementary File 4 Phosphoflow cytometry. **(A)** Exemplary raw data showing the gating of monocyte subsets and detection of phosphorylated p38 MAPK following incubation with different TLR ligands, or PBS as control. **(B)** Fold change in MFI as depicted in Figure 3D. **(C)** Statistical analysis of phosphoflow data shown in Figure 3D. Paired t-tests were performed on log-transformed MFI values of stimulated samples vs. PBS-incubated control samples. Obtained p values are shown alongside q values (two-stage step-up, Benjamini, Krieger, and Yekutieli; desired FDR 5%) and adjusted p values (Holm-Šídák method).
