# Supplementary material for: Monocyte biology conserved across species: Functional insights from cattle
Source: Front Immunol. 2022 Jul 29;13:889175. doi: 10.3389/fimmu.2022.889175 (PMC9373011; doi:10.3389/fimmu.2022.889175)
Supplement: Supplementary file 6 [file DataSheet_6.pdf]

Supplementary File 6

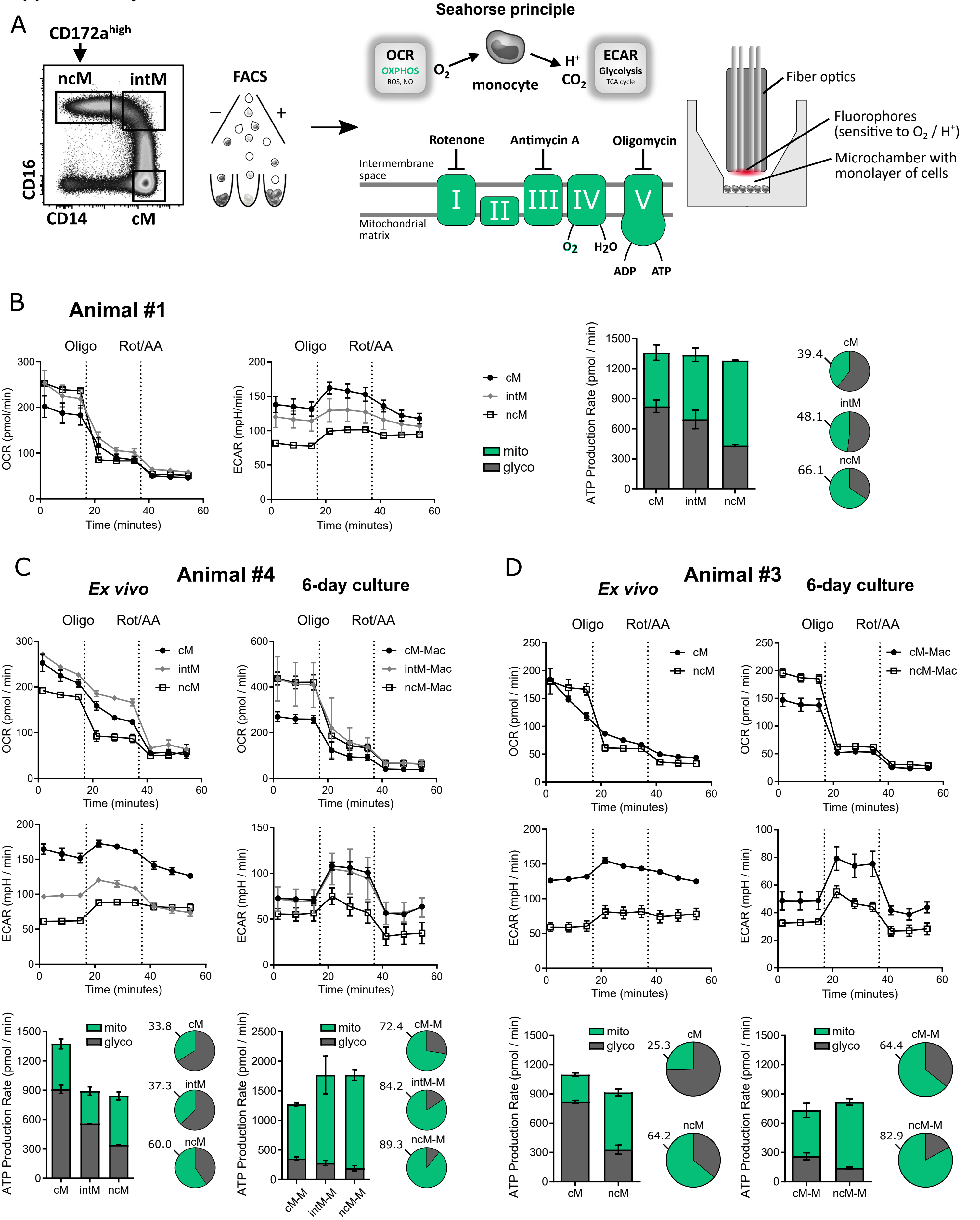

Supplementary File 6 Agilent Seahorse Assays. Bovine monocyte subsets were FACS-sorted and metabolic activity was analyzed by Agilent Seahorse XF technology and the XF Real-Time ATP Rate Assay**. (A)** Gating strategy as well as principle of Agilent Seahorse XF technology is illustrated. **(B-D)** OCR and ECAR traces as well as ATP production rates as absolute values (bar graphs) and relative values (pie charts) are shown for all three monocyte subsets (cM, intM, ncM) seeded in duplicates **(B+C)** or for cM and ncM seeded in triplicates **(D)**. Macrophages derived from monocyte subsets after six days of culture in the presence of M-CSF are shown in the right panels of C and D.
