# Supplementary material for: Monocyte biology conserved across species: Functional insights from cattle
Source: Front Immunol. 2022 Jul 29;13:889175. doi: 10.3389/fimmu.2022.889175 (PMC9373011; doi:10.3389/fimmu.2022.889175)
Supplement: Supplementary file 8 [file DataSheet_8.pdf]

Supplementary File 8A

Cell Ranger summary

CH4431

The analysis detected some issues. Details »

Alert	Value	Detail
Low Fraction Valid UMIs	74.6%	Ideal > 75%. This may indicate a quality issue with the Illumina R2 read for Single Cell 3' v1 or the R1 read for Single Cell 3' v2/v3 and Single Cell 5'. Application performance may be affected.

Estimated Number of Cells

6,697

Mean Reads per Cell	Median Genes per Cell
55,128	774

Sequencing

Number of Reads	369,197,587
Valid Barcodes	83.1%
Sequencing Saturation	57.6%
Q30 Bases in Barcode	95.7%
Q30 Bases in RNA Read	92.1%
Q30 Bases in UMI	95.7%

Mapping

Reads Mapped to Genome	90.8%
Reads Mapped Confidently to Genome	85.6%
Reads Mapped Confidently to Intergenic Regions	14.9%
Reads Mapped Confidently to Intronic Regions	32.3%
Reads Mapped Confidently to Exonic Regions	38.4%
Reads Mapped Confidently to Transcriptome	34.0%
Reads Mapped Antisense to Gene	1.9%

Cells

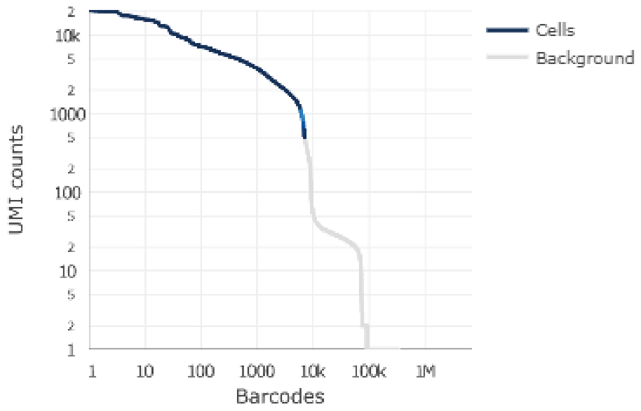

Estimated Number of Cells	6,697
Fraction Reads in Cells	85.5%
Mean Reads per Cell	55,128
Median Genes per Cell	774
Total Genes Detected	14,419
Median UMI Counts per Cell	1,951

Sample

Name	CH4431
Description	
Transcriptome	Bos_taurus.ARS-UCD1.2.dna.toplevel
Chemistry	Single Cell 3' v3
Cell Ranger Version	3.0.2

Supplementary File 8B

Cell Ranger summary

CH4432

The analysis detected some issues. Details »

Alert	Value	Detail
⚠ Low Fraction Valid UMIs	74.8%	Ideal > 75%. This may indicate a quality issue with the Illumina R2 read for Single Cell 3' v1 or the R1 read for Single Cell 3' v2/v3 and Single Cell 5'. Application performance may be affected.

Estimated Number of Cells

6,713

Mean Reads per Cell	Median Genes per Cell
58,464	817

Sequencing

Number of Reads	392,475,502
Valid Barcodes	86.4%
Sequencing Saturation	60.5%
Q30 Bases in Barcode	94.8%
Q30 Bases in RNA Read	90.2%
Q30 Bases in UMI	94.7%

Mapping

Reads Mapped to Genome	91.2%
Reads Mapped Confidently to Genome	85.7%
Reads Mapped Confidently to Intergenic Regions	13.1%
Reads Mapped Confidently to Intronic Regions	29.2%
Reads Mapped Confidently to Exonic Regions	43.5%
Reads Mapped Confidently to Transcriptome	39.6%
Reads Mapped Antisense to Gene	1.5%

Cells

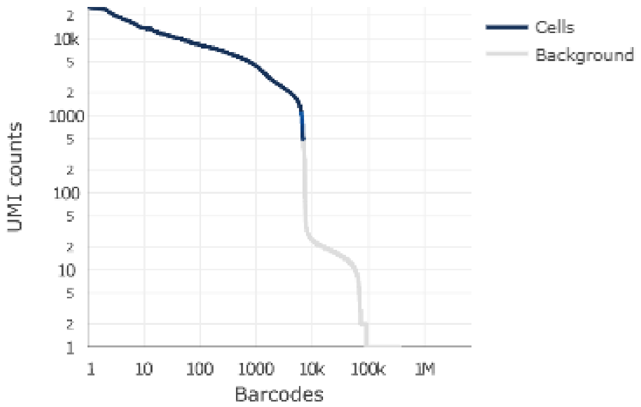

Estimated Number of Cells	6,713
Fraction Reads in Cells	93.3%
Mean Reads per Cell	58,464
Median Genes per Cell	817
Total Genes Detected	14,556
Median UMI Counts per Cell	2,201

Sample

Name	CH4432
Description	
Transcriptome	Bos_taurus.ARS-UCD1.2.dna.toplevel
Chemistry	Single Cell 3' v3
Cell Ranger Version	3.0.2
