# Supplementary material for: Monocyte biology conserved across species: Functional insights from cattle
Source: Front Immunol. 2022 Jul 29;13:889175. doi: 10.3389/fimmu.2022.889175 (PMC9373011; doi:10.3389/fimmu.2022.889175)
Supplement: Supplementary file 9 [file DataSheet_9.pdf]

Supplementary File 9

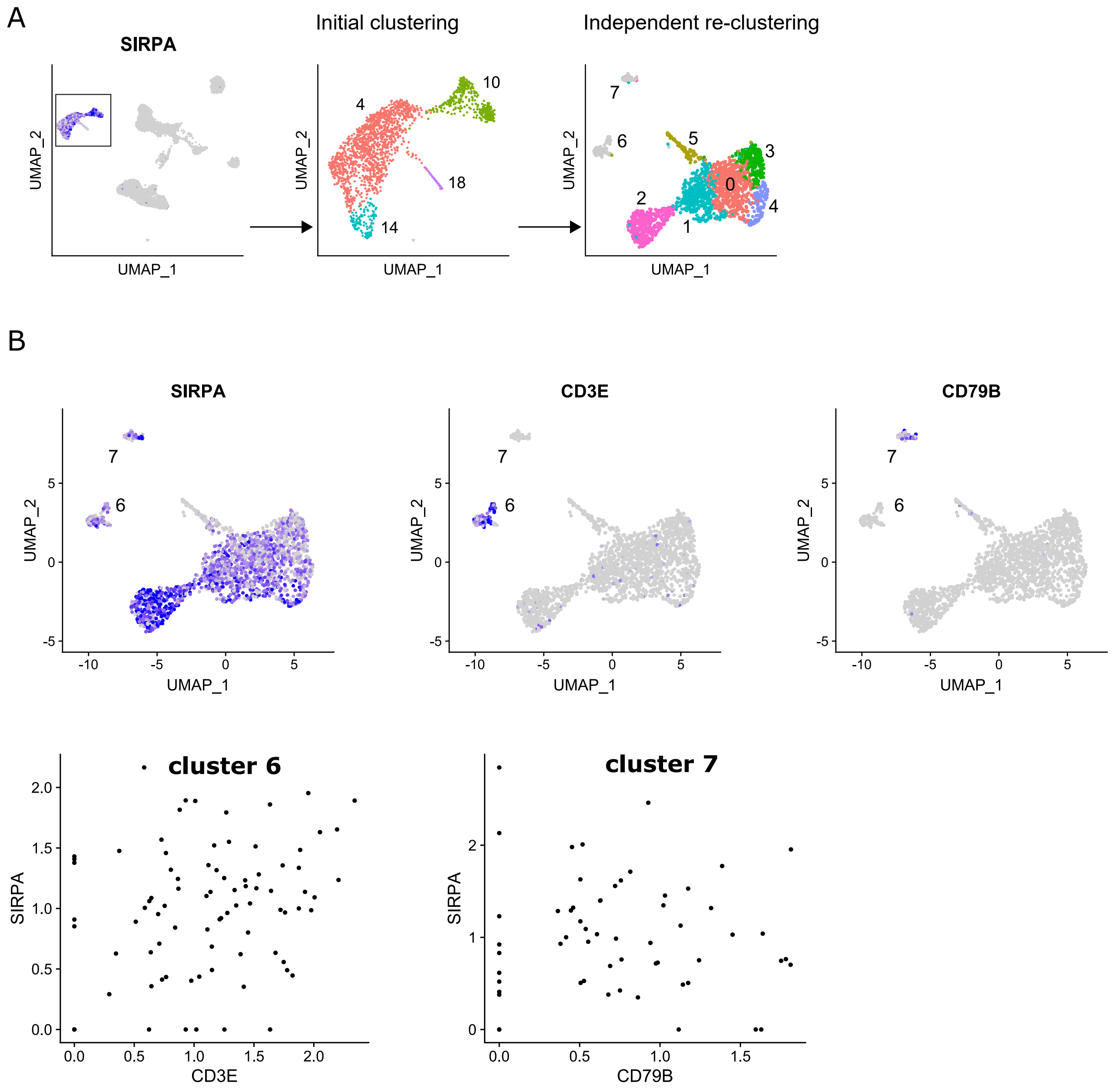

Supplementary File 9 Single-cell RNA-seq of bovine PBMC. **(A)** Initial clustering and independent re-clustering of *SIRPA*-expressing clusters within PBMC dataset. **(B)** Clusters 6 and 7, containing putative doublets co-expressing monocyte- and T-/B-cell markers, were excluded from further analysis.
